# Loss of CX3CR1 increases accumulation of inflammatory monocytes and promotes gliomagenesis

**DOI:** 10.18632/oncotarget.3730

**Published:** 2015-03-30

**Authors:** Xi Feng, Frank Szulzewsky, Alexan Yerevanian, Zhihong Chen, David Heinzmann, Rikke Darling Rasmussen, Virginia Alvarez-Garcia, Yeonghwan Kim, Bingcheng Wang, Ilaria Tamagno, Hao Zhou, Xiaoxia Li, Helmut Kettenmann, Richard M. Ransohoff, Dolores Hambardzumyan

**Affiliations:** ^1^ Department of Neurosciences at Cleveland Clinic, Cleveland, Ohio, USA; ^2^ Cellular Neurosciences, Max Delbrück Center for Molecular Medicine, Berlin, Germany; ^3^ Case Western Reserve University School of Medicine, Cleveland, Ohio, USA; ^4^ Department of Cardiology at Tübingen University School of Medicine, Tübingen, Germany; ^5^ Department of Stem Cell Biology and Regenerative Medicine, Cleveland, Ohio, USA; ^6^ Rammelkamp Center for Research, MetroHealth Center, Case Western Reserve University School of Medicine, Cleveland, Ohio, USA; ^7^ Department of Immunology at Cleveland Clinic, Cleveland, Ohio, USA; ^8^ Neuroinflammation Research Center, Cleveland Clinic, Cleveland, Ohio, USA

**Keywords:** CX3CR1/CX3CL1 signaling, glioblastoma, microglia, monocyte

## Abstract

The most abundant populations of non-neoplastic cells in the glioblastoma (GBM) microenvironment are resident microglia, macrophages and infiltrating monocytes from the blood circulation. The mechanisms by which monocytes infiltrate into GBM, their fate following infiltration, and their role in GBM growth are not known. Here we tested the hypothesis that loss of the fractalkine receptor CX3CR1 in microglia and monocytes would affect gliomagenesis. Deletion of *Cx3cr1* from the microenvironment resulted in increased tumor incidence and shorter survival times in glioma-bearing mice. Loss of Cx3cr1 did not affect accumulation of microglia/macrophages in peri-tumoral areas, but instead indirectly promoted the trafficking of CD11b^+^CD45^hi^CX3CR1^low^Ly-6C^hi^Ly-6G^−^F4/80^−/low^ circulating inflammatory monocytes into the CNS, resulting in their increased accumulation in the perivascular area. Cx3cr1-deficient microglia/macrophages and monocytes demonstrated upregulation of IL1β expression that was inversely proportional to Cx3cr1 gene dosage. The Proneural subgroup of the TCGA GBM patient dataset with high IL1β expression showed shorter survival compared to patients with low IL1β. IL1β promoted tumor growth and increased the cancer stem cell phenotype in murine and human Proneural glioma stem cells (GSCs). IL1β activated the p38 MAPK signaling pathway and expression of monocyte chemoattractant protein (MCP-1/CCL2) by tumor cells. Loss of Cx3cr1 in microglia in a monocyte-free environment had no impact on tumor growth and did not alter microglial migration. These data suggest that enhancing signaling to CX3CR1 or inhibiting IL1β signaling in intra-tumoral macrophages can be considered as potential strategies to decrease the tumor-promoting effects of monocytes in Proneural GBM.

## INTRODUCTION

The glioma subtype known as glioblastoma (GBM) is the most aggressive and most common primary brain tumor. Despite research advances, over 95% of patients succumb to their disease, resulting in a median survival time of only 14.6 months [1]. These dismal survival statistics highlight the urgent need for new approaches to GBM therapy. Analysis through The Cancer Genome Atlas (TCGA) initiative identified several subclasses of GBM, termed Proneural, Neural, Classical, and Mesenchymal, which are strongly associated with genomic abnormalities in platelet-derived growth factor receptor (PDGFR), isocitrate dehydrogenase 1 or 2 (IDH 1 or 2), epidermal growth factor receptor (EGFR), and neurofibromin 1 (NF1), respectively [2, 3]. Although initial studies showed that patients with a Proneural subtype had markedly better survival compared to the other subtypes [4], this phenomenon was conferred when glioma-CpG island methylator phenotype G-CIMP-positive tumors were excluded [5]. Differences among the subtypes are highlighted by the fact that they show differential responses to glioma cell-targeted standard therapies of various intensities, with better results achieved by more intense therapeutic regimes in all subtypes except Proneural [3].

Histological analysis of GBM revealed a heterogeneous cellular composition of neoplastic glioma cells and non-neoplastic cells, which form a tumor microenvironment composed of brain-resident microglia, infiltrating monocytes/macrophages, reactive astrocytes, endothelial cells, pericytes, neural stem/progenitor cells, and other immune cell infiltrates [6, 7]. Microglia and monocytes/macrophages constitute up to 30% of tumor mass in both human and murine GBM [7]. It has been demonstrated that microglia promote tumor growth [8], but to what extent tumor promotion is attributable to microglia versus monocytes/macrophages still remains unclear. It is also unclear to what extent GBM macrophages are derived from infiltrating cells from the blood circulation and what the ratio between microglia and monocyte/macrophages is. The importance of a nuanced understanding of the roles of microglia and monocyte/macrophages in GBM is highlighted by studies showing that these two cell types have dramatically different responses to injury [9, 10].

In addition to the invasive growth that prevents a complete surgical resection, the resistance of GBM to therapy is partially explained by two hallmark features of glioma stem cells (GSCs): radio-resistance and chemo-resistance. Experimentally, these cells can be enriched based on expression of a variety of surface markers [11, 12] or their ability to exclude Hoechst dye or DyeCycle violet (DCV) in a side population assay (SP) [13, 14]. GSCs reside in the perivascular area (PVA) in both human and murine gliomas [15, 16.] Thus, understanding the ways that microglia and infiltrating monocytes/macrophages interact with GSCs in the PVA may help to identify new therapeutic targets in the glioma microenvironment that can be combined with standard glioma therapies.

To study the role of microglia and monocytes/macrophages in GBM and their potential interactions with GSCs, we perturbed macrophage activation in tumors by genetically inactivating the CX3CR1/CX3CL1 signaling pathway. CX3CR1 is expressed by monocytes in the circulation and exclusively by microglia in the central nervous system (CNS) [17]. The ligand CX3CL1 is expressed by neurons and is the most abundantly expressed chemokine in the CNS. Accordingly, CX3CR1 regulates communications between neurons and microglia. The effects of CX3CR1 signaling on disease progression vary depending on the type of the CNS disease [18-21].

To study the functional role of CX3CR1/CX3CL1 signaling in GBM, we used a genetically engineered mouse model (GEMM) of adult PDGFB-driven gliomas, which is based on RCAS/Tv-a, a somatic cell-specific gene transfer system [22]. In terms of both gross pathology [23] and molecular profile, these tumors strongly resemble the human Proneural GBM subtype, which represents 30% of human GBM [3, 4]. As these mice are immunocompetent, this model is a suitable system to study interactions between immune and glioma cells. Here, we show that loss of CX3CR1/CX3CL1 signaling in tumor-associated microglia and monocytes results in an increased incidence of GBM formation with shorter tumor latency in glioma-bearing mice. While having no effect on microglial migration in peri-tumoral regions, loss of *Cx3cr1* did increase Ly-6C^hi^ “inflammatory” monocyte infiltration from the blood circulation into GBM, where they preferentially localized in perivascular areas. Loss of *Cx3cr1* results in a dose-dependent increase in IL1β expression in microglia and macrophages. This overexpression of IL1β in turn promotes glioma growth, induces activation of the p38 MAPK pathway, increases the GSC phenotype, and upregulates CCL2 expression, which correlates with greater monocyte infiltration. These data suggest that CX3CL1/CX3CR1 signaling, which is the most active chemokine-signaling system in the healthy CNS and is not expressed by GBM cells, may have potential as a therapeutic strategy to decrease monocyte infiltration into GBM. Our findings also imply that IL1β may be a therapeutic target for human Proneural GBM patients.

## RESULTS

### *Cx3cr1* deficiency results in increased tumor incidence and shorter survival times in GBM-bearing mice

**Figure 1:**
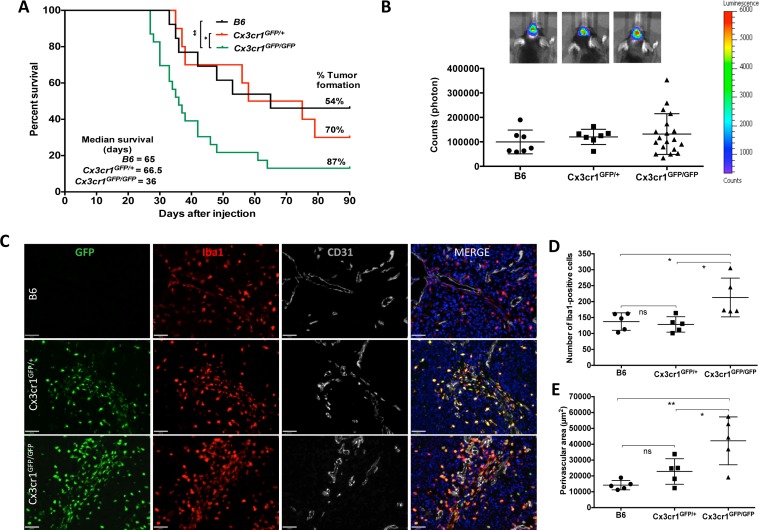
Homozygous deletion of *Cx3Cr1* in the tumor microenvironment increases the percentage of GBM formation and shortens tumor latency This leads to an increase in the total number of macrophages in tumors, which mainly accumulate in perivascular regions of GBM. **A**) Kaplan-Meier survival curves show that homozygous loss of *Cx3cr1* results in shortened survival of tumor-bearing mice compared to heterozygous loss of *Cx3cr1* or B6 mice (*Cx3cr1*^GFP/GFP^ vs. *Cx3cr1*^GFP/+^ MC p=0.0520, GBW *P < 0.05; *Cx3cr1*^GFP/GFP^ vs. B6 MC *P < 0.05, GBW **P<0.01; Log-rank (Mantel-Cox-MC) tests and Gehan-Breslow-Wilcoxon tests (GBW) were used). The tumor incidence (%) and median survival of tumor-bearing mice in Cx3cr1 homozygous, heterozygous knock-in and B6 mice are incorporated in the curves (n=13, 10, 23 for B6, *Cx3cr1*^GFP/+^ and *Cx3cr1*^GFP/GFP^, correspondingly). **B**) Representative BLI images of tumor-bearing mice and quantification of BLI in *Cx3cr1* homozygous, heterozygous knock-in and B6 mice at the end-point of survival curves showing no statistically significant differences in tumor size based on *Cx3Cr1* status. **C**) Brain tumor sections from *B6*, *Cx3cr1*^GFP/+^ and *Cx3cr1*^GFP/GFP^ mice with GFP (green), anti-Iba1 (red), and anti-CD31 (gray) and counterstained with DAPI (blue). Representative images demonstrate that *Cx3cr1*^GFP/GFP^ animals exhibit a higher number of Iba1-positive cells, which are mainly localized in perivascular areas of tumors. **D**) The number of Iba1-positive cells and perivascular areas were quantified in tumors from the three genotypes (n=5 animals per each genotype; left and right graph, correspondingly). Loss of two copies of *Cx3cr1* resulted in a statistically significant increase in the number of Iba1-positive cells and increased PVN area compared to the loss of one copy or wild-type (*p < 0.05), E) which reside in perivascular areas of GBM (one-way ANOVA with Tukey's multiple comparisons test, *p < 0.05 and **p < 0.01, respectively).

Microglia and monocyte infiltration and function are partially regulated by chemokines, which act on chemokine receptors such as CX3CR1. As CX3CR1 signaling has been implicated in both microglial activation and migration, we decided to study the role of CX3CR1 in gliomagenesis. For these studies, we used mice in which the *Cx3cr1* gene was inactivated following germline insertion of the green fluorescent protein (GFP) gene, such that heterozygous mice *Cx3cr1^GFP/+^* expressed the GFP reporter in cells that retained receptor function, whereas homozygous *Cx3cr1^GFP/GFP^* cells were labeled with GFP but lacked *Cx3cr1*expression (i.e. *Cx3cr1^GFP/GFP^* equals *Cx3cr1^−/−^*) [24]. We transplanted freshly dissociated cells from GBM generated in *Gli-luciferase*;*Nestin-Tv-a*;*Ink4-Arf^−/−^*;*pten ^fl/fl^* (referred to as *NiG* mice) into *B6* (strain control or *Cx3cr1^+/+^*), *Cx3cr1^GFP/+^* and *Cx3cr1^GFP/GFP^* mice. Homozygous loss of *Cx3cr1* resulted in a significant decrease in tumor latency and in increased tumor incidence (Fig. 1A). A trend toward increased tumor incidence was also observed in heterozygous GBM-bearing mice (Fig. 1A). The survival curve summarizes the tumor incidence and median survival times of tumor-bearing mice from the three different genotypes. Next we evaluated whether the shorter survival times of tumor-bearing mice in the *Cx3cr1*-deficient background might result from tumor location or size at the end-point of survival. All tumor-bearing mice independent of background demonstrated similar neurological signs including lethargy, grooming, and weight loss of 15% or more, which were used as criteria to determine the end-point. As transplanted tumor cells also had a Gli-luciferase reporter, we visualized tumors by BLI during tumor growth (data not shown) and at the end-point of survival *in vivo* and *ex vivo*. The size of tumors generated in *B6*, *Cx3cr1^GFP/+^* and *Cx3cr1^GFP/GFP^*, as determined by BLI, was not significantly different between groups (Fig. 1B). *Ex vivo* BLI imaging of tumor-containing whole brains also showed no significant differences in tumor location in the three genotypes (data not shown).

### CX3CL1 expression is decreased in both murine and human GBM tissue

CX3CL1 was abundantly expressed in naïve brains of *NiG* mice, while mRNA expression was significantly less in murine GBM samples and freshly sorted GBM cells (Fig. S1A). Assessment of REMBRANDT data also revealed that CX3CL1 mean expression intensity was decreased in human GBM samples compared to non-tumor control samples (Fig. S1D). CX3CL1 protein was abundantly expressed in naïve brain but was undetectable in GBM tissue, freshly sorted or cultured GBM cells (Fig. S1B and S1C). These data suggest that tumor cells do not express detectable levels of CX3CL1 protein and that mRNA levels are low compared to the naïve brain, which could be partially attributable to the known phenomenon of significantly decreased numbers of neurons in GBM tissue.

### In gliomas, ***Cx3cr1*** deficiency increases density of microglia/macrophages, which are preferentially localized in perivascular areas

CX3CR1 signaling is implicated in the survival of microglia and monocytes/macrophages. We thus determined the effect of CX3CR1 deficiency on macrophage density in GBM. Quantification of CX3CR1 (based on GFP-expression) and ionized calcium-binding adapter molecule 1 (Iba1) double-positive cells from tumors generated in *Cx3cr1^GFP/+^* and *Cx3cr1^GFP/GFP^* mice revealed that over 95% of CX3CR-positive cells were also positive for Iba1 (data not shown, Fig. 1C). Based on these data, we then used Iba1 to quantify the density of macrophages in tumors generated in the three genotypes. Homozygous loss of *Cx3cr1* resulted in a statistically significant increase in the density of macrophages in tumors (Fig. 1C and 1D). Iba1/CX3CR1 double-positive cells were mainly located in the PVA (Fig. 1C). Quantification of PVA, based on area occupied by Iba1-positive cells, in tumors generated in the three genotypes demonstrated statistically significant increases in the sizes of PVA due to increased area occupied by macrophages in GBM in *Cx3cr1*-deficient hosts (Fig. 1E). There were no genotype-dependent differences in either total vessel area or average vessel size in either tumors or the surrounding brain tissue (Fig. S2A and S2B). These data suggest that the increases in PVA and the number of macrophages observed in tumors from *Cx3cr1^GFP/GFP^* mice are not due to increased angiogenesis.

### *Cx3cr1* deficiency results in a significant increase in Ly-6C^hi^ “inflammatory” monocyte infiltration into GBM

**Figure 2:**
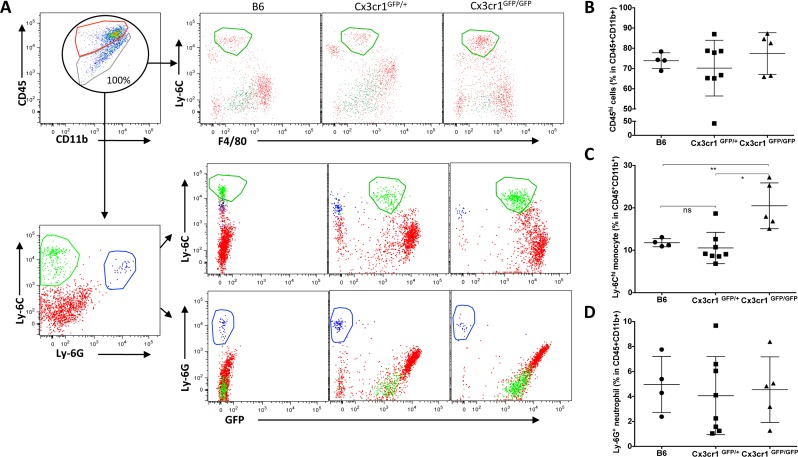
Loss of *Cx3Cr1* results in a significant increase of Ly-6C “inflammatory” monocyte infiltration into GBM **A**) Representative dot plots that are gated on CD11b^+^CD45^+^ cells, with red and green circles defining CD11b^+^CD45^hi^ (blood-derived macrophages) and CD11^+^CD45^lo/int^ (resident brain microglia). Total population of CD11b^+^CD45^+^ is considered as 100%, and they are further gated on Ly-6C and F40/80 positivity for tumors from the three genotypes (upper panel). The CD45^+^CD11b^+^ population is further gated for Ly-6C and Ly-6G positivity to distinguish monocytes from neutrophils and further gated for GFP (CX3CR1), which shows that while inflammatory monocytes are positive for GFP, neutrophils are negative. **B**) Dot plots represent the percentage of CD45^hi^ population in the total CD11b^+^CD45^+^ population of tumors from the three genotypes. Although there is a trend towards an increase in CD45^hi^ in tumors from *Cx3cr1*^GFP/GFP^ mice, it does not reach statistical significance (n=4, 8 and 5 individual tumors for *B6*, *Cx3cr1*^GFP+^ and *Cx3cr1*^GFP/GFP^, respectively). Tumor sizes were chosen close to the end-point of survival to ensure similar sizes in the three different genotypes. **C**) Dot plots represent the percentage of Ly-6C^hi^ monocytes in the total CD11b^+^CD45^+^ population in tumors from the three different genotypes (each dot corresponds to one animal). A one-way ANOVA with Tukey's multiple comparisons test was performed and demonstrated that there was a statistically significant increase in the percentage of Ly-6C^hi^ monocytes in tumors from *Cx3cr1*^GFP/GFP^ animals compared to B6 or *Cx3cr1*^GFP+^ animals (*p < 0.05 and **p < 0.01, correspondingly). **D**) Dot plots represent the percentage of Ly-6G^+^ neutrophils in tumors from the three different genotypes showing that loss of one or both copies of *Cx3cr1* had no impact on neutrophil infiltration into GBM.

Increased density of macrophages in stromal *Cx3cr1*-deficient tumors can be caused by either local expansion of microglia or by increased infiltration of monocytes from the blood circulation, possibly accompanied by expansion of this population. To test for increased monocyte density, we analyzed CD11b and CD45 expression in similar-sized tumors at the end-point of survival in each of the three genotypes using flow cytometry. Previously, characterization of tumor-associated microglia and macrophages by flow cytometry identified that, although both resident microglia and blood macrophages have high CD11b expression, they show differential expression levels for the CD45 surface antigen, with microglia being low and blood macrophages being high [25, 26]. After setting the total CD45^+^CD11b^+^ population to 100%, we then examined differences in the percentages of CD45^hi^ (hematogenous) cells versus CD45^low^ cells (microglia) (Fig. 2A). While there were no significant differences observed in the percentage of CD45^hi^ cells in CX3CR1-negative tumors (Fig. 2B), we found a significant enrichment of CD11b^+^ CD45^hi^ Ly-6C^hi^ Ly-6G^−^ F4/80^−/low^ immature or inflammatory monocytes in tumors generated in *Cx3cr1*-deficient mice as compared to tumors in heterozygous *Cx3cr1* or wild-type (WT) mice (Fig. 2C). This population was CX3CR1^low^ as previously reported [31]. Ly-6G staining confirmed the monocytic lineage of these cells and also showed that there were no genotype-dependent differences in neutrophil infiltration (Fig. 2D). Next, we evaluated whether the phenomenon of increased monocyte infiltration is also observed in the brains of naïve *Cx3cr1*-deficient mice compared to heterozygous *Cx3cr1* or WT mice. There were no significant differences in the percentages of CD45^hi^ or infiltrating monocytes in *Cx3cr1*-deficient mice compared to heterozygous *Cx3cr1* or WT mice (Fig. S3), suggesting that this is GBM-specific. Taken together, these data in Figures 1 and 2 show increased GBM infiltration by inflammatory monocytes and a higher density of tumor-associated macrophages in mice lacking CX3CR1.

**Figure 3:**
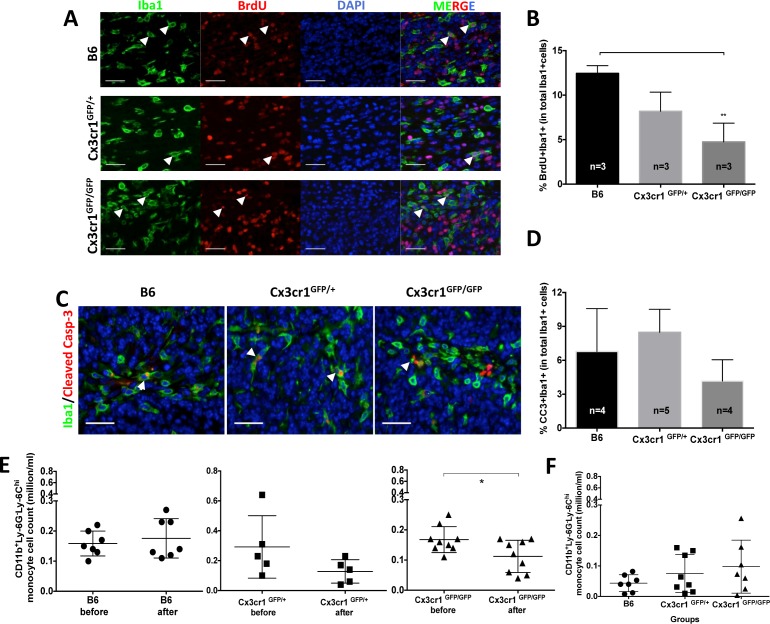
The increased number of inflammatory monocytes in *Cx3cr1*-deficient background is due to their increased infiltration from the blood and not to local proliferation or decreased cell death **A**) Representative images of brain tumor sections from B6, *Cx3cr1*^GFP+^ and *Cx3cr1*^GFP/GFP^ mice were stained with anti-Iba1 (green), anti-BrdU (red), and counterstained with nuclear DAPI (blue). **B**) Quantified bar graphs from A showing that there was no statistically significant difference in the number of BrdU^+^Iba1^+^ cells in tumors from the three different genotypes. **C**) Representative images of brain tumor sections from B6, *Cx3cr1*^GFP+^ and *Cx3cr1*^GFP/GFP^ mice were stained with anti-Iba1 (green), anti-Cleaved Caspase-3 (red), and counterstained with nuclear DAPI (blue). **D**) Quantified bar graphs from C showing that there was no statistically significant difference in the number of BrdU^+^ Cleaved Caspase-3^+^ cells in tumors from the three different genotypes. For B and D, one-way ANOVAs with Tukey's multiple comparisons tests were performed and demonstrated that there were no statistically significant differences observed in the number of BrdU^+^Iba1^+^ and BrdU^+^ Cleaved Caspase-3^+^ cells in the three genotypes. **E**) Dot plots representing the number of CD11b^+^Ly-6C^hi^ Ly-6G^−^ inflammatory monocytes were analyzed from the blood of the same mice before and 20 days after tumor cell transplantation. There was a significant decrease in Ly6C^hi^ inflammatory monocytes in *Cx3cr1*^GFP/GFP^ mice, suggesting increased infiltration to GBM. **F**) Dot plots represent the number of CD11b+Ly-6Chi Ly-6G^−^ inflammatory monocytes when mice were sacrificed at the end-point of survival. There was a significant difference in CD11b^+^Ly-6C^hi^ Ly-6G^−^ inflammatory monocytes when the three genotypes were compared and they all showed a reduction compared to naïve mice before the transplant, see E. A paired t-test was used in Figure 6E and one-way ANOVA with Tukey's multiple comparisons test was performed for F. Scale bars for A and C represent 50 μm.

To test whether the local proliferation of monocytes, macrophages and microglia contributes to their increased accumulation in PVA, we performed a BrdU incorporation experiment with two pulses every 6 hours (at 10 mg/kg body weight) for 12 hours before sacrificing tumor-bearing animals of the three genotypes and quantifying Iba1^+^BrdU^+^ cell numbers. We did observe small but significant differences in the proliferation rates of Iba1-positive cells between tumors generated in WT (B6) and *Cx3cr1*-deficient mice (*Cx3cr1*^GFP/GFP^ mice) (Fig. 3A and 3B). Next we examined whether decreased levels of apoptosis of Iba1^+^ cells in PVA could contribute to their increased numbers in tumors generated in *Cx3cr1*-deficient mice. We stained tumors from the three genotypes for cleaved-caspase-3 and Iba1. We did not observe significant differences in cleaved-caspase-3^+^BrdU^+^ cell numbers in any of the three genotypes (Fig. 3C and 3D). Next we analyzed whether the amount of Ly-6C^hi^ monocytes in the blood changes over the course of disease progression in host *Cx3cr1*-deficient tumor-bearing mice, potentially contributing to their increased density in tumors. The number of CD11bv Ly-6C^hi^Ly-6G^−^ inflammatory monocytes in the blood was analyzed before mice were subjected to stereotactic surgeries for tumor cell transplantation and then blood collection was repeated 20 days post-surgery, when mice did not yet show signs of tumors. The density of inflammatory monocytes, however, was significantly decreased in *Cx3cr1*-deficient mice with tumors (Fig. 3E), a decrease which might be caused by increased infiltration into the CNS. In end-point of survival experiments, all mice showed decreases in inflammatory monocytes as compared to their original blood levels (data not shown) and there were no significant differences between groups (Fig. 3F). These data suggest that the increased density of inflammatory monocytes in *Cx3Cr1*-deficient mice is due to their increased infiltration into the CNS from the blood circulation during disease progression.

### Loss of *Cx3cr1* leads to a dose-dependent increase in IL1β expression by microglia/macrophages

**Figure 4:**
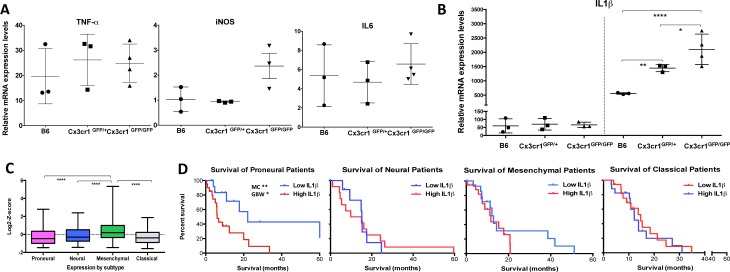
Loss of ***Cx3cr1*** results in a dose-dependent increase in IL1β expression by CX3CR1-positive cells IL1β expression is up-regulated in human GBM and levels of expression correlate with Proneural patient survival. **A**) Relative mRNA expression dot plots from freshly FACS-sorted CD11b^+^CD45^+^CX3CR1^+^ cells from the three genotypes showed no differences in *TNF-α, iNOS* or *IL6* levels, **B**) while a significant dose-dependent increase was observed in IL1β expression levels. All samples were normalized to the mRNA levels of β-actin and are presented as fold-change compare to naïve brains. A one-way ANOVA with Tukey's multiple comparisons test was performed and demonstrated that there was a statistically significant dose-dependent increase in IL1β expression (*p < 0.05 (*Cx3cr1*^GFP^) and **p < 0.001(*Cx3cr1*^GFP/GFP^) compared to B6). **C**) Relative IL1β mRNA expression in the four GBM subtypes of the TCGA data set. A Newman-Keuls multiple comparisons test was used (****p < 0.0001; n=118, 75, 139, 128 for Proneural, Neural, Mesenchymal and Classical, respectively). **D**) Kaplan-Meier survival curves for the Proneural, Neural, Mesenchymal and Classical subtypes. High and Low are defined as +/−1 Standard Deviation for each subtype. Log-rank (Mantel-Cox-MC) tests and Gehan-Breslow-Wilcoxon tests (GBW) were used to determine p values. The only significant difference in survival was observed in Proneural patients (*p < 0.05 MC and **p < 0.01 GBW).

To gain insight into the tumor-promoting effects of CX3CR1-deficient cells in the tumor microenvironment, the expression levels of TNF-α, iNOS, IL6 and IL1β, which each have altered expression due to loss of CX3CR1 in different CNS diseases [18, 20], were examined by quantitative RT-PCR. CD11b^+^CD45^+^Ly-6G^−^ and CD11b^+^CD45^+^Ly-6G^−^GFP^+^ cells were sorted from tumors generated in *B6*, *Cx3cr1^GFP/+^* and *Cx3cr1^GFP/GFP^* mice, correspondingly; RNA was isolated and analyzed for TNF-α, iNOS, IL6, and IL1β expression. While no significant changes were observed between the three genotypes for *TNF-α, iNOS*, or *IL6* by qPCR (Fig. 4A), we confirmed a significant dose-dependent increase (when one or both copies of the *Cx3cr1* gene were removed) in IL1β expression. We excluded the possibility of constitutive differences in IL1β expression in naïve brains from the three different genotypes (Fig. 4B). We analyzed the TCGA data set [2] for relative IL1β mRNA expression in human GBM subgroups (Fig. 4C), as our murine tumors strongly resemble the human Proneural GBM subtype, and found that the Proneural group with high IL1β expression showed significant survival differences compared to patients with Proneural tumors with low IL1β expression (Fig. 4D). IL1β expression was not associated with survival in the other GBM subtypes.

### IL1β increases glioma cell growth/viability and enhances the glioma stem cell phenotype

**Figure 5:**
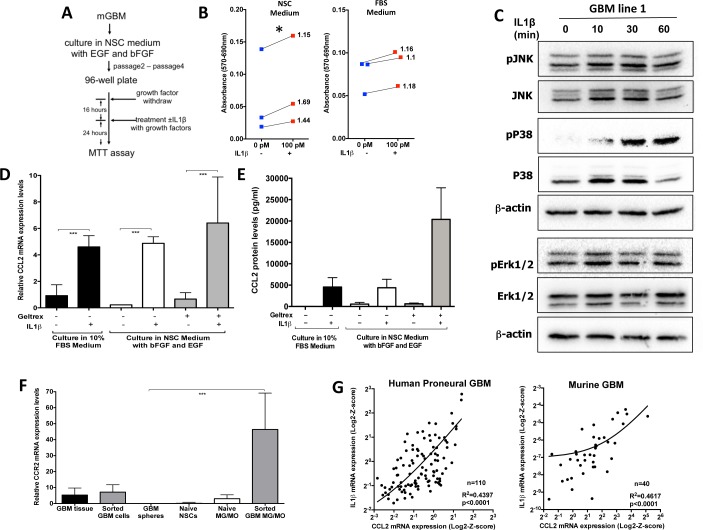
IL1β treatment increases glioma cell growth/viability, activates p38/JNK and NF-κB pathways in GSC-enriching medium, and leads to activation of CCL2 expression **A**) Illustration of the steps for the MTT assay. **B**) Quantification of the MTT assay performed on GBM spheres, which were cultured in NSC (left) or 10% FBS (right) with or without IL1β 24h later. These data show that while there was no significant increase in the growth in FBS, there was a significant difference in NSC conditions. We used three independent primary tumors to derive three independent glioma lines for two conditions. Blue squares show the growth of the original tumor and they are connected to red squares that represent the growth in response to IL1β; values on the graphs represent fold increase in growth/viability in response to IL1β compare to control growth. An unpaired t-test was used to determine the fold change compare to control, * *p* < 0.05, 95% CI (1,33; 1,69). **C**) Immunoblot analysis was performed to examine the p38 MAPK, JNK and ERK1/2 signaling pathways 0, 10, 30 and 60 minutes after 100pM IL1β treatment. The Western blots shown were performed on the GBM 1 and GBM 2 lines (cultured in Geltrex). Similar results were obtained on GBM sphere 3. **D**) Relative mRNA expression dot plots for CCL2 from glioma cells cultured in different conditions with or without 100pM IL1β treatment, showing that IL1β treatment significantly increased CCL2 mRNA expression, independent of culture conditions. **E**) The CCL2 protein levels measured by ELISA in the supernatant showed an increase in response to 100pM IL1β treatment. **F**) Relative mRNA expression dot plots for CCR2 expression from different cell types showing that CCR2 was not expressed in cultured glioma cells when grown in NSC conditions. A one-way ANOVA with Tukey's multiple comparisons test was performed, ***p < 0.001 **G**) The right graph shows correlation with linear regression from 110 proneural GBM samples for IL1β and CCL2 RNA expression from the TCGA database. A statistically significant positive correlation was found between IL1β and CCL2 RNA expression r=0.63, p<0.0001. Linear regression R2=0.4397, *p* < 0.001. The left graph shows correlation with linear regression from 40 murine PDGF-B-driven GBM samples for IL1β and CCL2 RNA expression. A statistically significant positive correlation was found between IL1β and CCL2 RNA expression r=0.68, p<0.001. Linear regression R2=0.4617, *p* < 0.001.

Next we examined whether IL1β can promote tumor growth in our murine Proneural GBM model. We generated primary cultures from PDGFB-driven gliomas generated in NiG mice. Freshly isolated tumor cells were cultured in two different conditions: the first condition was chosen to enrich for GSCs (serum-free neural stem cell medium supplemented with EGF and bFGF), while the second condition was chosen to enrich the main/bulk tumor population (10% FBS in DMEM medium). We performed MTT assays for the dose response to recombinant IL1β and showed that the most effective dose for growth was 100pM (data not shown). While 24h incubation with IL1β showed significant increases in growth/viability in GSC-enriching conditions (Fig. 5B, left panel), IL1β mediated no growth advantage for cells cultured in FBS-containing medium (Fig. 5B, right panel). These data suggest that IL1β signaling selectively affects cells grown in GSC-enriching conditions. Next we evaluated IL1R1 expression and showed that both mRNA and protein are expressed in GBM cells (Fig. S4A and S4B). In response to IL1β binding to IL1R1, a complex sequence of combinatorial phosphorylation and ubiquitination events results in activation of nuclear factor κB (NF-κB) signaling and the JNK and p38 mitogen-activated protein kinase pathways, which cooperatively induce the expression of canonical IL1β target genes (such as *IL-6, CXCL8, CCL2, COX-2, IκBα, IL1α, IL1β, MKP-1*) via transcriptional and post-translational modifications (see detailed discussion in review [27]). Next we examined what downstream targets were activated in response to IL1β treatment in GSCs. IL1β treatment induced activation of the p38 MAPK pathway in all four mouse GSC lines tested, but not extracellular signal-regulated kinase (ERK) (Fig. 5C, Fig. S5). p38 MAPK has been shown to play important roles in glioma invasion, progression and therapy resistance [28]. CCL2 has been reported to be one of the canonical IL1β target genes and is a known chemoattractant ligand for the CCR2 receptor, which is expressed in inflammatory monocytes and has been shown to be important for exit of monocytes from the bone marrow to the blood circulation and subsequent trafficking into the site of inflammation [29]. Next we examined whether CCL2 is upregulated by IL1β treatment in cultured glioma cells. There was significant upregulation of CCL2 mRNA (Fig. 5D) and protein expression levels (Fig. 5E) independent of culture conditions, whereas CCR2 was preferentially expressed by inflammatory monocytes and macrophages (Fig. 5F). Since our data showed that IL1β regulates CCL2 mRNA expression in glioma cells, we examined whether there was a correlation between IL1β and CCL2 mRNA expression levels in human GBM subtypes (from TCGA database) and murine GBM. We observed a statistically significant positive linear association between IL1β and CCL2 mRNA expression levels both in human Proneural and murine GBM (Fig. 5G). There was a significant positive linear association in the Classical subtype, but not in the Neural and Mesenchymal subtypes (Fig. S6). Together, these data help explain the increased inflammatory monocyte infiltration into GBM generated in the *Cx3cr1*-deficient background. Since *Cx3cr1*-deficient inflammatory monocytes and monocyte-derived macrophages express higher levels of IL1β compared to heterozygous *Cx3cr1* and WT tumors, they induce increases in CCL2 expression by glioma cells, which in turn causes the recruitment of more CCR2-positive inflammatory monocytes into the tumor, as shown in Fig. 2A and 2B. IL1β from inflammatory monocytes has a paracrine effect on glioma cells and induces increased levels of CCL2 in tumor cells, which in turn has a paracrine effect on inflammatory monocytes, resulting in increased infiltration.

### *Cx3cr1* loss via increased expression of IL1β leads to an enhanced glioma stem cell phenotype *in vitro* and *in vivo*

**Figure 6:**
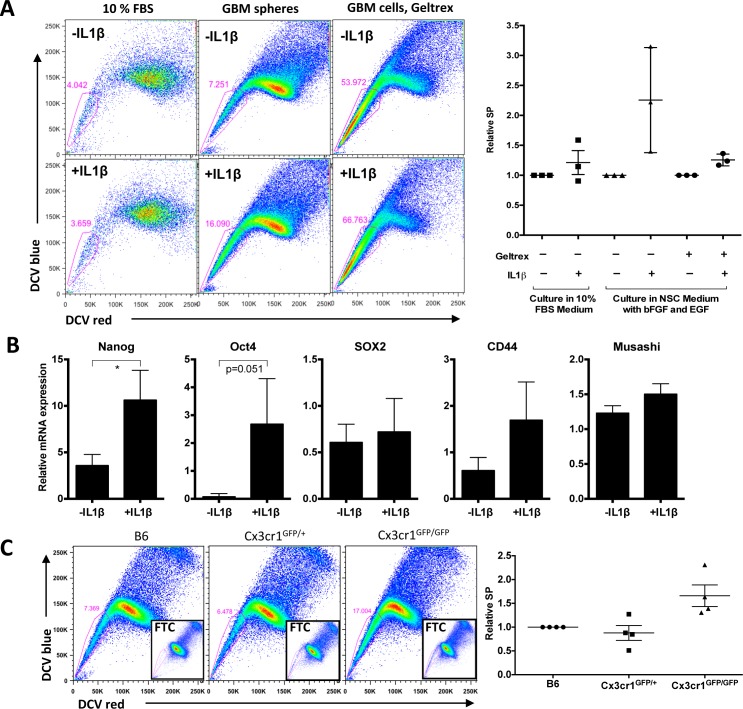
IL1β treatment enhances a stem cell phenotype *in vitro*, which correlates with *Cx3Cr1* loss *in vivo* **A**) SP analysis of freshly dissociated tumor cells cultured in 10% FBS medium, in NSC medium as spheres, and cultured in NSC medium like monolayer. Cells were treated with or without 100pM IL1β for 24h. The right graph shows relative SP from three independent glioma lines in three different conditions. **B**) qPCR data for relative mRNA expression of Nanog, Oct4, Sox2, CD44 and Musashi in GBM cells cultured in NSC medium with geltrex as a monolayer. Data represent average value of three independent cell lines. Error bars represent SD. *p < 0.05 by unpaired t-test. **C**) SP analysis of GBM samples generated in B6, *Cx3cr1*^GFP+^ and *Cx3cr1*^GFP/GFP^ mice. Inserts show FTC-treated samples. The right graph represents relative SP from for 4 independent tumors per genotype.

We observed an increase in PVA, where glioma stem cells are localized, in host *Cx3cr1*-deficient tumors. We therefore examined whether IL1β-treated glioma cells that were maintained in GSC-enriching medium would show an increase in the glioma stem cell population *in vitro*. We used the SP assay, which is a well-accepted method of identifying glioma stem cells in murine and human models. Freshly isolated cells from tumors were cultured in two different conditions, GSC-enriching conditions and the main/bulk-enriching (10% FBS in DMEM medium) condition. IL1β significantly increased the percentage of SP cells in three independent tumor-derived primary cells in GSC-enriching medium, while no increase was observed in cells that were cultured in serum (Fig. 6A). Therefore, we tested whether IL1β treatment could induce expression of stem-like cell markers. Nanog and Oct4 expression were increased, whereas Sox2 expression levels were not increased (Fig. 6B). This observation is attributable to the fact that Sox2 is both a GSC marker and is also associated with the Proneural molecular subtype in human [30], so its expression can be extended beyond GSCs in mouse tumors as well (data not shown). CD44 and Musashi expression were not increased (Fig. 6B). Together, these data suggest that monocytes and macrophages can drive elements of a stem cell phenotype in PDGFB-induced gliomas via IL1β expression.

It was also of interest to determine whether the effect of IL1β on the glioma stem cell phenotype occurs in human GSCs. In view of our finding that high IL1β expression correlated with poor survival only in human Proneural GBM, we treated two human Proneural GBM GSCs with human IL1β for two weeks. By using a limited dilution assay, we showed that self-renewal of both GSCs7-11 and 827 [31, 32] were increased by IL1β compared to control (Fig. S7).

To confirm whether this effect of IL1β on the stem cell phenotype is *Cx3cr1*-dependent, we analyzed tumors generated in *B6*, *Cx3cr1^GFP/+^* and *Cx3cr1^GFP/GFP^* mice at the end-points of survival. We observed large variability in the % SP (7-45%) in tumors from all three genotypes, which was associated with the time of survival of tumor-bearing mice. We normalized SP from tumors in *Cx3cr1^GFP/+^* and *Cx3cr1^GFP/GFP^* mice to B6 tumors based on the time of tumor development and represented it as relative SP, which was much higher in tumors from *Cx3cr1^GFP/GFP^* compared to *Cx3cr1^GFP/+^* and B6 (Fig. 6C). These data suggest that the *in vitro* effects of IL1β on stemness are driven by *Cx3cr1* deficiency *in vivo*. Since it has been shown that increased SP in gliomas correlates with unregulated CD44 expression and radiation resistance in the same murine GBM model, we examined whether CD44 expression is increased in PVA area when tumors from *Cx3cr1^GFP/GFP^* mice are compared to *Cx3cr1^GFP/+^* and B6. We did observe an increase in CD44-positive areas in tumors generated in *Cx3cr1^GFP/GFP^* mice compared to *Cx3cr1^GFP/+^* and B6 (Fig. S8).

### Loss of *Cx3cr1* has no impact on microglia/macrophage accumulation in peri-tumoral areas or on tumor growth in organotypic slice cultures

**Figure 7:**
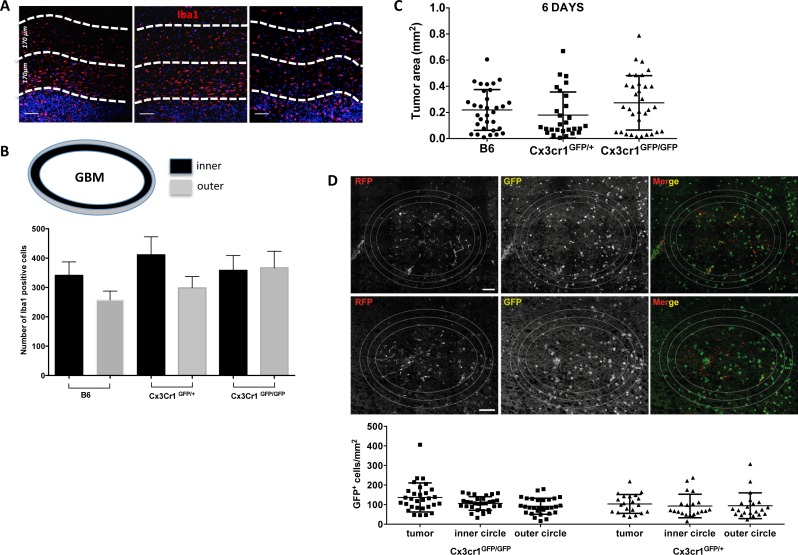
*Cx3cr1* deficiency has no impact on microglial accumulation at the close edge of GBM and has no impact on tumor growth in organotypic slice cultures **A**) Representative images of Iba1 staining in tumors from the three genotypes. The white broken-lines in the images are drawn to show the close and distant edge of the tumors (170 μm each) and DAPI-positive nuclear density was used as guide to separate the tumor from the peri-tumoral area. **B**) Quantified bar graphs for the entire peri-tumoral areas that were constructed from a series of 20x images that cover the entire peri-tumoral area (n=7, 6, 7 for B6, *Cx3cr1*^GFP+^ and *Cx3cr1*^GFP/GFP^, correspondingly). **C**) Quantification of tumor areas in organotypic brain slice cultures from the three genotypes at 6 days post-tumor cell inoculation are presented as dot plots. **D**) Representative images of GFP-positive cells from slices generated from *Cx3cr1*^GFP+^ and *Cx3cr1*^GFP/GFP^ mice. The white lines in the images are drawn to show the inner and outer layers of the tumors (80 μm each). The quantified numbers of GFP-positive cells in the inside, inner and outer layer of tumors are presented as dot blots. A one-way ANOVA with Tukey's multiple comparisons test was performed and demonstrated that there were no statistically significant differences observed in tumor volumes in the three genotypes (C and D). Scale bar represents 50 μm for A and 150 μm for D.

Loss of one copy of the *Cx3cr1* gene has been shown to reduce accumulation of microglia (by 50%) and delay optic nerve glioma development [33]. Thus we decided to evaluate microglia/macrophage accumulation in peri-tumoral areas in tumors from the three genotypes by quantifying the total number of Iba1^+^ cells in two concentric peri-tumoral layers of tissue (Fig. 7A and 7B). There were no significant differences in the numbers of Iba1^+^ cells in the first and second peri-tumoral layers in GBM tissue from *B6* and *Cx3cr1^GFP/+^* mice compared to the tumors in *Cx3cr1^GFP/GFP^* mice. These data suggest that loss of *Cx3cr1* does not affect accumulation of microglia in peri-tumoral areas. In order to assess the functional consequences of CX3CR1 deficiency on microglial migration and the effect it has on tumor growth, we generated gliomas *ex vivo* in naïve organotypic slice cultures generated from B6, *Cx3cr1^GFP/+^* and *Cx3cr1^GFP/GFP^* mice as described in Supplemental Figure 9. This method allowed us to investigate the role of microglial CX3CR1 on tumor growth. There were no significant differences in the size of the tumors at 6 days post-tumor cell inoculation. Next, we fixed the organotypic slices, sectioned them into 8μm sections, and quantified GFP^+^ microglial density in tumors generated *ex vivo* in slices from the three genotypes. Quantification of GFP^+^ microglia inside of the tumor and inner and outer peri-tumoral areas showed no significant differences in microglia density whether one or two copies of *Cx3cr1* were deleted. These data suggest that the effect of *Cx3cr1* loss on tumor growth and increased stem cell-like cell phenotype *in vivo* is driven by increased infiltration of inflammatory monocytes and their increased expression of IL1β.

## DISCUSSION

In recent years, accumulating evidence from several tumor types has shown the importance of non-neoplastic cells in the tumor microenvironment for the maintenance of cancer growth and response to therapy. In gliomas, microglia and macrophages together represent the most abundant non-neoplastic cell types, which are present in both low- and high-grade gliomas. While in low-grade gliomas, the main population of macrophages is resident brain microglia [33], we show here that in high-grade gliomas, one finds a mixture of resident microglia, inflammatory monocytes and macrophages. Over the last decade, our knowledge of the role of microglia in gliomas has evolved significantly [8], but we have yet to determine the mechanism by which bone marrow-derived monocytes infiltrate into GBM, their fate following infiltration, or their role in GBM growth and how different they are from microglia. These are all valid questions considering major differences in the origin and life span of these cells. Monocytes are myeloid-derived cells that belong to the mononuclear phagocyte system. They arise from hematopoietic stem cells in the bone marrow, are released into the bloodstream, and colonize peripheral organs in normal and inflammatory conditions [34], where they differentiate into macrophages or dendritic cells and have multiple functions in homeostasis and innate and acquired immunity. Microglia, on the other hand, originate from yolk sac progenitors that migrate into the brain in the early stages of development (E10.5) and are maintained locally via self-renewal [35].

In this study we abrogated CX3CR1/CX3CL1 signaling in both microglia and inflammatory monocytes and studied the effects on glioma growth and on the behavior of both cell populations. First, we showed that the loss of CX3CR1 signaling in the host microenvironment results in increased GBM formation and shortened tumor latency. One correlative study showed that single nucleotide polymorphisms (SNPs heterozygous-CX3CR1-I249) in the human *Cx3cr1* gene resulted in decreased microglial infiltration and longer survival of GBM patients that underwent surgery [36], although, whether the post-surgery tissue contained inflammatory monocytes or only microglia was not investigated. Further studies are warranted to investigate the role of the CX3CR1-I249 polymorphism based on genetic subtypes of glioma. Another report using GL261 murine glioma cells demonstrated that loss of *Cx3cr1* in the host microenvironment has only a slight effect on survival of tumor-bearing mice and no differences were observed in the density of tumor-infiltrated immune cells or microglial accumulation [37]. These differences can be partially explained by differences in the properties of glioma cells that have been used in transplant experiments. In contrast to the GL261 model, data from the experiments using GEMM of low-grade gliomas showed that decreased expression of CX3CR1 resulted in delayed tumor growth and decreased accumulation of microglia in tumors; most importantly the authors reported that over 95% of macrophages in low-grade gliomas are microglia [33]. Differences in all the above-mentioned studies and our current study show that the role of CX3CR1/CX3CL1 can vary depending on the grade and subtype of glioma. This can be partially attributed to the fact that GBM subtypes show activation of different core signaling pathways. Further studies are warranted to determine whether monocyte infiltration also occurs in other GBM subtypes. Here we demonstrated that loss of *Cx3cr1* in the host microenvironment resulted in increased accumulation of bone marrow-derived Ly6-C^high^ “inflammatory” monocytes in GBM, in contrast to microglia in low-grade gliomas.

Our finding of increased monocyte infiltration as a result of *Cx3cr1* loss in GBM shows similarity with recent data in myelorestoration, where the CX3CR1/CX3CL1 axis controls the release of Ly-6C^high^monocytes from marrow such that in *Cx3cr1^GFP/GFP^* mice, Ly-6C ^high^ monocytes accumulated less rapidly in the marrow but recovered faster in the blood, resulting in more accumulation into the spleen [38]. Increased inflammatory monocyte accumulation in GBM as a result of *Cx3cr1* loss is further supported by data showing that human CX3CL1 (also known as fractalkine) antagonizes transendothelial migration and chemotaxis of freshly isolated human monocytes induced by CCL2. Our PDGFB-driven murine GBM significantly up-regulated levels of CCL2 mRNA and protein expression (unpublished data from our laboratory). This would help explain the fact that tumor cells up-regulate CCL2 to attract monocyte infiltration into GBM, while on the other hand they do not express CX3CL1. Increased monocyte infiltration as a result of *Cx3cr1* loss in our GBM model is further supported by a study showing formation of leaky and immature microvessels lacking CX3CR1-positive smooth muscle-like cells, extracellular collagen, and basement membrane laminin in *Cx3cr1^GFP/GFP^* but not in *Cx3cr1^GFP/+^* mice, when matrigel plugs were implanted [39]. We also demonstrated that loss of CX3CR1 results in dose-dependent increases in IL1β expression, which in turn upregulates CCL2 expression and recruits more monocytes. Thus when the loss of *Cx3cr1* resulted in further increases in CCL2 expression, we observed greater “inflammatory” monocyte infiltration into GBM, mainly localizing in the PVA. Similar results with increased IL1β resulting from loss of *Cx3cr1* were shown in two Alzheimer's disease mouse models and other CNS diseases [18, 20]. IL1β was also shown to increase the self-renewal of intestinal epithelial stem cells [40]. Recently, we have demonstrated the existence and complexity of the PVA in the PDGF subgroup of human gliomas and in our PDGF-driven mouse gliomas. The PVA of PDGF-driven human and mouse gliomas is a complex structure, which contains a treatment-resistant, nestin-positive cancer stem-like cell population [16]. In gliomas, it has been shown that IL1β in combination with TGFβ increases the neurosphere-forming ability and tumorigenicity of the LN-229 glioma cell line, although the mechanism was not elucidated [41]. Our data demonstrate that IL1β in GSCs induces activation of p38 MAPK and enhances the GSC phenotype *in vitro* and *in vivo*, which seems to be *Cx3cr1*-dependent. These effects of IL1β can partially explain the tumor-promoting effect of CX3CR1 in our mouse model of GBM. In human GBM, high expression of IL1β was also correlated with shorter survival only in Proneural GBM patients. IL1β treatment also increases the stem cell frequency in two proneural human GSC lines. Only in Proneural and murine GBM did we observe significant positive correlations between expression levels of CCL2 and IL1β.

Our data demonstrate that while increasing monocyte infiltration and tumor-promoting effects, loss of *Cx3cr1* has no impact on microglia/macrophage accumulation in peri-tumoral areas *in vivo* or in organotypic slice cultures of gliomas *ex vivo*. The use of organotypic slice cultures allowed us to generate gliomas in a monocyte-free environment, where we demonstrated that loss of *Cx3cr1* in microglia had no impact on glioma growth. These results suggest that the tumor-promoting effect produced by the loss of *Cx3cr1* is a result of infiltrating inflammatory monocytes from the blood circulation. This observation is further supported by data showing that loss of *Cx3cr1* has no impact on infiltration of other immune cells from the blood, which include neutrophils and dendritic cells (DCs). Though it has previously been proposed that CNS DCs can arise from transformed microglia *in vitro* in which microglia up-regulate CD11c in the presence of GM-CSF [42], in our GBM model we did not observe up-regulation of CD11c in microglia or in the bone marrow-derived CD45^hi^CD11b^+^ population.

Taken together, our results provide insights into the differences in CX3CR1 signaling in microglia versus bone marrow-derived “inflammatory” monocytes, which includes migration and tumor-promoting effects. In light of these data, the terms “Tumor-associated macrophages (TAMs)” that are widely used in the glioma literature should be used very carefully, as this is a highly heterogeneous population that includes resident brain microglia, infiltrating blood monocytes, and macrophages, showing very different functions. Based on our data, CX3CR1 signaling has differential functions in bone marrow-derived inflammatory monocytes versus resident microglia. These data also confer the importance of studying the biology of inflammatory monocytes in the context of GBM as well as the molecules that they secrete to promote tumor growth. IL1β is one example that we identified in these studies, but this is likely only one of many remaining to be identified.

## MATERIALS AND METHODS

### Mice

Animals were housed in the Cleveland Clinic Biological Resource Unit. All experimental procedures were approved by the Institutional Animal Care and Use Committee of the Cleveland Clinic (Animal Protocol 2013-1029). Mice (6-10 weeks old) were used in all experiments. We used age and sex as criteria to equally distribute mice from different genotypes for all experiments. *Gli-Luciferase; Nestin-tv-a;Ink4a-Arf*^−/−^;*pten^fl/fl^* mice were generated as previously described. *Cx3cr1^GFP+^*, *Cx3cr1^GFP/GFP^* mice were generated from heterozygous breeding pairs, backcrossed for more than 10 generations to B6 [24].

### Cell cultures and transfection

DF-1 cells were purchased from ATCC, Manassas, VA. Cells were grown at 39°C according to instructions from ATCC. Transfections with RCAS-PDGFB-HA, RCAS-PDGFB-SV40-RFP and RCAS-CRE were performed using a Fugene 6 transfection kit (Roche, Mannheim, Germany) according to manufacturer's instructions. Transfection efficacies were assessed by Western blotting and PCR.

### Generation of tumors

Transgenic mice (*Gli-Luciferase; Nestin-tv-a;Ink4a-Arf*^−/−^;*pten^fl/fl^* referred to as NiG) were anesthetized with intraperitoneal injections of a mixture of ketamine (0.1 mg/g) and xylazine (0.02 mg/g). One microliter of 4×10^4^ cell suspension containing either RCAS-PDGF-B-HA or RCAS-PDGFB-RFP + RCAS-Cre transfected (1:1 ratio) DF1 cells was delivered using a 30-gauge needle attached to a Hamilton syringe and stereotactic fixation device (Stoelting, Wood Dale, IL). Locations were determined according to a mouse brain atlas [43]. Cells were injected into the right frontal striatum with the following coordinates from bregma: AP (anterior) 1.0 mm, ML (medial/lateral) 1.0 mm, and DV (dorsal/ventral) 2.0 mm. Mice were monitored carefully and were sacrificed if they displayed lethargy or head tilt due to tumor burden.

### Orthotopic glioma generation

The same procedure was used as described above, except 2.5×10^4^ of freshly-dissociated tumor cells were injected into the frontal striatum of recipient animals. Two or three tumors were used for obtaining single cells for orthotopic glioma generation in *B6*, *Cx3cr1^GFP+^*, and *Cx3cr1^GFP/GFP.^* The GBM grade of donor tumors was validated by H&E staining. The tumor cell injections into *B6*, *Cx3cr1^GFP+^*, and *Cx3cr1^GFP/GFP^* recipients were performed on the same day with the same cells.

### Bioluminescence imaging (BLI)

Mice were anesthetized with 3% isoflurane and hair covering the head was shaved before retro-orbital injection with 75mg/kg body weight of D-luciferin (30mg luciferin/ml dH_2_O at 2.5 μl per gram mouse weight). One minute after injection of D-luciferin, images were acquired for 8 min with an IVIS 100 (Xenogen) imaging system. A photographic image was taken onto which the pseudocolor image representing the spatial distribution of photon count was projected. A circular region (1.4cm diameter) of interest (ROI) covering the tumor region was defined and used to quantify the bioluminescent signals in all experiments. All representative images were formatted using the same maximum and minimum threshold parameters.

### Tissue processing

Animals were anesthetized with a mixture of ketamine (0.15 mg/g) and xylazine (0.02 mg/g), perfused with ice-cold Ringer's solution, and sacrificed. Brains were removed and processed according to the different applications. For H&E validation and immunohistochemistry, brains were fixed in 10% neutral buffered formalin for 72 hours at RT, processed in a tissue processer (Leica, Wetzlar, Germany), embedded in paraffin, sectioned (5 μm), and slide-mounted. For immunofluorescent staining, brains were fixed in 4% PFA overnight at 4°C, sunk in 30% sucrose (dissolved in PBS) for 48 hours at 4°C, embedded in Optimal Cutting Temperature (OCT, Tissue-Tek, Torrance, CA) compound, sectioned (8 μm), slide-mounted, and stored at −80°C.

### Immunoflurescent staining (IF)

5 μm and 8 μm coronal sections were used for paraffin-embedded and frozen sections in all histological studies. The following antibodies were used at the stated dilutions: rabbit polyclonal anti-Iba1, 1:100 (Wako Pure Chemicals, Osaka Japan); rabbit polyclonal anti-GFP, 1:100 (Life Technologies, Grand Island, NY); rabbit polyclonal anti-Olig2, 1:250 (Chemicon, Temecula CA); mouse monoclonal anti-PCNA, 1:2000 (DAKO, Glostrup, Denmark); and rat monoclonal anti-CD31, 1:100 (Dianova, Germany), rabbit polyclonal cleaved caspase 3, 1:100 (Cell signaling, Danvers, MA), rat anti-BrdU, 1:100 (BIO-RAD, Hercules, CA). For IF staining, secondary antibodies conjugated to different Alexa-Fluor dyes (488 nm, 555 nm, 647 nm from Life Technologies, Grand Island, NY) at a dilution of 1:500 in PBS/2% BSA were applied. For nuclear counterstaining, DAPI was used (Sigma-Aldrich, St. Louis, MO).

### BrdU incorporation

At the end point of survival, tumor-bearing mice were given i.p. two pulses every 6 hours (at 10 mg/kg body weight, Sigma-Aldrich, St. Louis, MO) for 12 hours before they were sacrificed.

### Quantification of IF

For quantification of Iba1-positive cells and CD31 staining (Fig. 3), five to ten images (20x) of perivascular regions were taken per mouse brain using CD31 staining as a reference. The total numbers of Iba1-positive cells as well as CD31-positive areas were assessed using ImageJ. For Iba1-positive cell quantification in peri-tumoral areas (Fig. 6), adjacent 10x images (1600 × 1200 pixels, 300 dpi) were taken and recomposed using Photoshop software (Adobe System Incorporated); an outline of the tumor border was then determined by DAPI staining and two layers (200 pixels or 240 μm width) outside of this border were then determined and named as the inner and outer layers. Cell numbers were counted with ImageJ and normalized to a 1600 × 200 pixel area (1360×170 μm^2^).

### Flow cytometry

For FACS analysis, brains were digested in 0.25% Trypsin/EDTA without phenol red at 37°C for 10 minutes (GBM) or 30 minutes (naïve brains, without cerebellum). Digestion was stopped by adding 2 volumes of RPMI medium containing 10% FBS. Cells were passed through a 40 μm cell strainer, centrifuged and resuspended in 30% Percoll (GE Healthcare, Princeton, NJ) solution, and laid above a 70% Percoll layer (diluted in RPM medium with 1% FBS). Cells were separated by centrifuging at 800xg for 30 minutes at 4°C. Cells from the 30%/70% Percoll interphases were collected and washed with FACS buffer (DPBS with 0.5% BSA) and blocked with 100 μl of 2x blocking solution (2% FBS, 5% normal rat serum, 5% normal mouse serum, 5% normal rabbit serum, 10 μg/ml 2.4g2 anti-FcR and 0.2% NaN_3_ in DPBS) on ice for 30 minutes. Cells were then stained with various antibodies (see the list of antibodies in Supplemental Table 1A) on ice for 30 minutes and washed with FACS buffer. For blood analysis, blood samples were taken via tail vein bleeding, lysed with RBC lysis buffer (Biolegend, San Diego, CA), washed with FACS buffer, counted with a hemocytometer, and stained using the same protocol before analyzing. The list of antibodies is included in Table S1A. All data were collected on a LSR II Fortessa (BD Biosciences, San Jose, CA) and analyzed using FlowJo 9 software (Tree Star Inc., Ashland, OR).

**For tumor cell sorting**, brains were digested for 15 minutes (GBM) or 30 minutes (naïve brains, without cerebellum) at 37°C in 5 ml of freshly prepared digestion solution (0.94 mg/ml papain (Worthington), 0.48 mM EDTA, 0.18 mg/ml N-acetyl-L-cysteine and 0.06mg/ml DNase I (Sigma-Aldrich, St. Louis, MO) diluted in Earl's Balanced Salt Solution (EBSS)). Two ml of Ovomucoid solution (0.71 mg/ml dissolved in Neural Stem Cell (NSC) Basal Medium) was used to terminate the digestion. Cells were washed in NSC Basal Medium and passed through a 40 μm cell strainer before sorting.

**For immune cell sorting**, cells were further washed with HBSS, resuspended in 30% Percoll solution (prepared with RPMI medium with 1% FBS), and placed under a HBSS aqua phase. Cells were centrifuged (low acceleration and no brake) at 950xg for 20 minutes at 4°C. Pelleted cells were washed with FACS buffer before sorting. Staining was performed as described above.

### RNA extraction and real-time PCR

Tumors and naïve brains from tumor-bearing or control animals were removed after transcardiac perfusion with ice-cold Ringer's solution and snap frozen and stored at −80°C until RNA extraction. RNA from tumor tissue or freshly sorted cells was extracted using an RNeasy Plus Mini kit (QIAGEN, Valencia, CA) and from naïve brains using an RNeasy Lipid Tissue Mini kit (QIAGEN, Valencia, CA) according to the manufacturer's instructions.

RNA concentrations were measured with a NanoDrop spectrophotometer and samples were stored at −80ºC. cDNA was synthesized from total RNA using the SuperScript III First-Strand Synthesis System (Life Technologies, Grand Island, NY). Real-time PCR (quantitative PCR) was performed using a FastStart SYBR Green Master mix (Roche Applied Science, Mannheim, Germany) according to manufacturer's instructions. Amplification was performed in the Applied Biosystems 7500 Fast Real-Time PCR System (Life Technologies). Primer sequences are shown in Supplemental Table 1B. β-actin was used as an internal control and the ΔΔCT method was used to calculate changes in fold expression.

### Tumor dissociation and primary cell cultures

PDGF-driven tumors were dissociated as described in the flow cytometry section above. Briefly, tumors were dissected and collected in basal neural stem cell culture medium. Five ml of digestion solution per tumor was used to digest tissues. Cells were consecutively washed and resuspended to obtain a single cell suspension. For tumorsphere cultures, cells were seeded at 50×10^4^ cells/ml and grown in mouse neurosphere medium (Stem Cell Technologies, Vancouver, Canada) supplemented with 10ng/ml hEGF, 20 ng/ml basic-hFGF, 1 mg/ml Heparin, and NSC Proliferation Supplements. Fresh medium was added to the cultures every 48 hours before treatment. For primary monolayer cultures, cells were cultured in high glucose DMEM supplemented with 10% FBS; medium was changed every 2 days. For IL-1β treatment and MTT assays, cells were grown as adherent monolayer cultures on Geltrex-coated tissue culture plasticware (Life Technologies, Grand Island, NY), which were prepared according to manufacturer's instructions. This approach for *in vitro* experiments provided better-controlled and more reproducible results compared to when cells are grown as neurospheres. When GSCs are grown as neurospheres, the size of the neurospheres is highly variable and can greatly impact the availability of exogenous IL1β for cells in the core of spheres. It becomes especially crucial when time points for collecting samples after adding exogenous IL1β are short.

### Organotypic slice cultures

P14-P16 *Cx3cr1*GFP/GFP, *Cx3cr1^GFP/+^*, and *B6* littermate mice were decapitated and brains were removed and placed in ice-cold phosphate-buffered saline (PBS) under sterile conditions. The forebrain was dissected from the brainstem and glued (using cyanoacrylate glue) onto a glass block and cut in the coronal plane into 250 μm sections with a vibratome (Microm HM 650 V, Thermo Scientific, Waltham, MA). Brain slices were transferred onto 0.4 μm polycarbonate membranes in the upper chamber of a transwell tissue insert (Falcon model 3090, Becton Dickinson, Franklin Lakes, NJ), which was inserted into a 6-well plate (Falcon model 3502, Becton Dickinson, Franklin Lakes, NJ). Thereafter, brain slices were incubated in 1 ml of culture medium per well containing DMEM supplemented with 10% heat-inactivated fetal bovine serum (Atlanta Biological, Norcross, GA), 0.2 mM glutamine, 100 U/ml penicillin, and 100 mg/ml streptomycin (medium-1). After overnight equilibration of the brain slices in medium-1, this was exchanged for cultivation medium (medium-2). Medium-2 contained 25% heat-inactivated horse serum, 50 mM sodium bicarbonate, 2% glutamine, 25% Hank's balanced salt solution, 1 mg/ml insulin (all from Life Technologies, Grand Island, NY), 2.46 mg/ml glucose (Braun Melsungen, Germany), 0.8 mg/ml vitamin C (Sigma-Aldrich, St. Louis, MO), 100 U/ml penicillin, 100 mg/ml streptomycin (Sigma-Aldrich, St. Louis, MO), and 5 mM Tris in DMEM (Life Technologies, Grand Island, NY).

### Tumor cell injections into cultured brain slices

One day after cutting, 5000 cultured RCAS-PDGFB-SV40-RFP tumor cells in a volume of 0.1μl were injected into the slices using a Hamilton syringe mounted to a micromanipulator. An injection canal was formed that reached 150 μm deep into the 250 μm-thick slice. The needle was then retracted by 50μm, leaving an injection cavity of approximately 50μm. The cell suspension was slowly injected over 30 seconds and subsequently the syringe was slowly pulled out in 10μm incremental steps over 60 seconds. To ensure identical experimental conditions, gliomas were always inoculated into the same area. Tumor sizes were determined 6 and 12 days post-injection by fluorescence microscopy (Axiovert 135, Carl Zeiss, Jena Germany) and the fluorescent area was evaluated with Fiji ImageJ.

### Re-cutting of cultured slices

For analysis of microglial invasion into tumor areas, slices were fixed 4 days post-injection of tumor cells with 4% PFA for 1 hour and subsequently stored in 30% sucrose until further use. Fixed slices were mounted on plain-cut blocks of Tissue-Tek O.C.T. (Sakura, Tokyo, Japan), covered with more Tissue-Tek O.C.T., and subsequently cut into 10μm sections using a Leica CM1950 Cryostat (Leica Microsystems, Wetzlar, Germany). Confocal images were prepared using a Leica TCS SPE confocal microscope (Leica Microsystems) and evaluated using Adobe Photoshop and Fiji ImageJ.

### Immunoblot analysis

Antibody against actin was purchased from Santa Cruz Biotechnologies, Santa Cruz, CA. Cells were harvested and lysed in a Triton-containing lysis buffer (0.5% Triton X-100, 20mM HEPES (pH 7.4), 150 mM NaCl, 12.5 mM β-glycerophosphate, 1.5 mM MgCl_2_, 10 mM NaF, 2 mM dithiothreitol, 1 mM sodium orthovanadate, 2mM EGTA, 1mM phenylmethylsulfonyl fluoride and complete protease inhibitor cocktail from Roche). Cell lysates were then separated by 10% SDS-PAGE, transferred to Immobilon-P membranes (Millipore), and subjected to immunoblotting. Antibodies against phosphorylated JNK, p38 (Thr180/Tyr182), and Erk1/2 (Thr202/Tyr204) were purchased from Cell Signaling, Danvers, MA. Quantification of western blots was performed using ImageJ. Intensity values for phosphorylated proteins were normalized per totals, which were first normalized per actin of each blot. Four independent primary mouse tumor-derived GSCs were used for western blots.

### Limiting Dilution Assay (LDA)

LDA was performed in 96-well plates. Briefly, dissociated cells were seeded at a range of 1–100 cells per well with or without bFGF and EGF and then 400 pM IL1β was added every 3 days. Cells were incubated at 37°C for 2 weeks. At the time of quantification, each well was examined for the formation of tumor spheres. Stem cell frequency and *p*-values were calculated using a web-based tool “ELDA” (extreme limiting dilution analysis), which is available on the Walter and Eliza Hall Institute of Medical Research web site (http://bioinf.wehi.edu.au/software/elda/).

The 827 and GSC7-11 cells were generously provided by Drs. Jeognwu Lee and Erik Sulman. Both cell lines display characteristics of the Proneural subtype, as revealed by RNA-seq, by centroid analysis (827) [31], and by microarray analysis using the unsupervised algorithm (GSC7-11) [32]. GSCs were cultured in neurobasal media with N2 and B27 supplements (0.5× each; Invitrogen) and human recombinant bFGF and EGF (25 ng/ml each; R&D Systems) (NBE condition).

### MTT assay

Cells from murine GBM tissues were cultured in 10% FBS containing medium (DMEM) or neural stem cell medium. 10k cells were seeded into each well in a 96-well plate. 24 hours later, medium was changed to 1% FBS DMEM or neural stem cell medium without bFGF or EGF. Cells were starved for 16h before IL1β treatment, followed by 24 hours of 100pM of IL1β (401-ML-005/CF R&D, Minneapolis, MN) diluted in 1% FBS DMEM medium or complete neural stem cell medium (with EGF and FGF) for FBS cultures and neural stem cell cultures, respectively. MTT assays (TOX1-1KT) were performed according to the manufacturer's instructions (Sigma-Aldrich, St. Louis, MO).

### Side population analysis (SP) by FACS

SP analysis was performed as described previously. Briefly, after tumor dissociation, cells were incubated at 37ºC with (controls) or without (samples) Fumitremorgin C (Sigma-Aldrich, St. Louis, MO), followed by staining with Dyecycle Violet (Life Technologies, Grand Island, NY) at 37ºC for 90 minutes. Cells were washed and stained with propidium iodide (PI) before analyzing. Signals were excited by the violet laser (407 nm) on LSR Fortessa. DCV-red and DCV-blue signals were collected through 670/30 nm and 450/50 nm filters, respectively. For *in vitro* experiments, cells were incubated with or without 100 pM IL1β, collected, and analyzed for SP as described above.

### TCGA analysis

Expression values and patients survival data for each gene of interest were obtained from the MSKCC computational biology cancer genomics portal-cBioPortal (http://www.cbioportal.org/cgx/index.do) which has annotated TCGA data [44, 45]. (Study: Glioblastoma multiforme, TCGA Provisional, mRNA Expression z-Scores (microarray), accessed in June 2014. Tumor subtype classification was previously described [3].

### Statistical analysis

Graphs were created using GraphPad Prism 6 (GraphPad Software Inc., La Jolla, CA) and were analyzed using an unpaired parametric two-tailed t-test, assuming equal standard deviations. One-way ANOVA was used in experiments having more than one group to compare to controls. Test details are included in appropriate figure legends; (*) *P* < 0.05; (**) *P* < 0.01; (***) *P* < 0.001; (no asterisks) not significant.

## SUPPLEMENTARY MATERIALS, TABLES, FIGURES


