# Supplementary material for: Loss of CX3CR1 increases accumulation of inflammatory monocytes and promotes gliomagenesis
Source: Oncotarget. 2015 Mar 30;6(17):15077–94. doi: 10.18632/oncotarget.3730 (PMC4558137; doi:10.18632/oncotarget.3730)
Supplement: Supplementary file 1 [file oncotarget-06-15077-s001.pdf]

Loss of CX3CR1 increases accumulation of inflammatory monocytes and promotes gliomagenesis

Supplementary Material

Mouse chemokine arrays: Freshly sorted tumor cells (labeled and sorted based on RFP expression), cultured GBM cells (cultured in neurosphere conditions with bFGF and EGF until passage 2 (Lee et al., 2006)), GBM tissue, which contained tumor cells plus tumor-associated stroma, and naïve brains, which were used as a positive control. Total of 300µg protein per each sample, were used to perform chemokine arrays, which contained 25 different capture antibodies, including CX3CL1. Membranes immobilized with the 25 captured antibodies, including CX3CL1, were purchased from R&D Systems and processed according to the manufacturer's instructions.

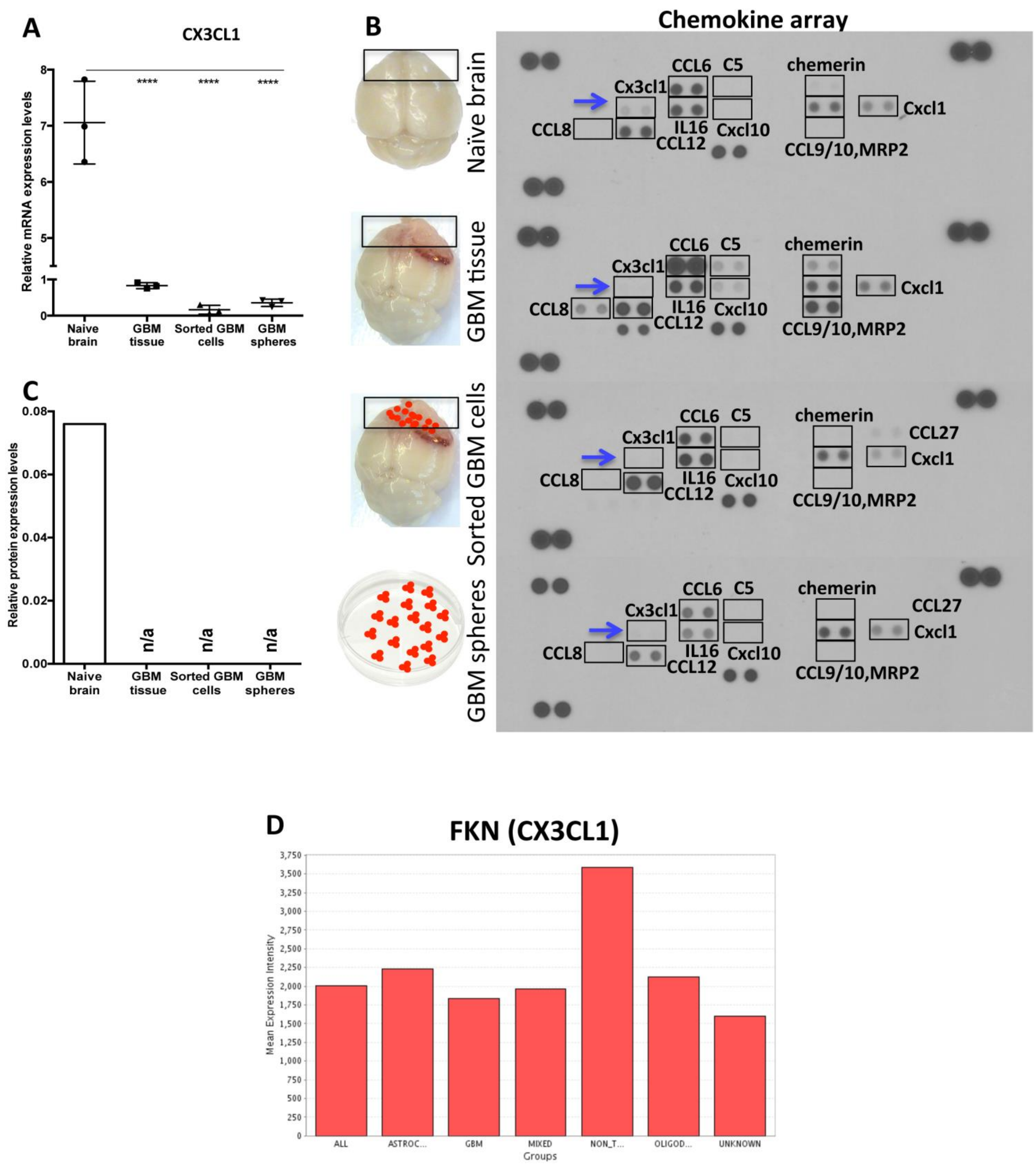

Figure S1: CX3CL1 RNA and protein expression is down-regulated in GBM. A) Relative mRNA expression levels of CX3CL1 in naïve brain, PDGFB-driven GBM tissue, sorted GBM cells and cultured GBM spheres driven from NiG mice (n=3 for all the groups). The mRNA levels were normalized to mRNA levels of β-actin. There was a significant decrease in CX3CL1 mRNA levels when GBM tissue,

sorted GBM cells, and GBM spheres were compared to naïve brain (Dunnett’s multiple comparison test, **** $p<0.0001$). **B)** Chemokine arrays were performed using 300µg protein from naïve brain, GBM tissue, freshly-FACS sorted GBM cells (based on RFP), and cultured GBM cells at passage 2. **C)** Quantification of relative protein expression of CX3CL1 identified from chemokine arrays. **D)** Bar graph of FKN RNA expression in non-tumor and in different types and grades of gliomas from REMBRANDT**.** ALL: All glioma (n=454), ASTROC: Astrocytoma (n=148), GBM: Glioblastoma (n=228), MIXED: Mixed (n=11), NON_T: Non-tumor (n=28), OLIGOD: Oligodendroglioma (n=67). UNKNOWN (n=67).

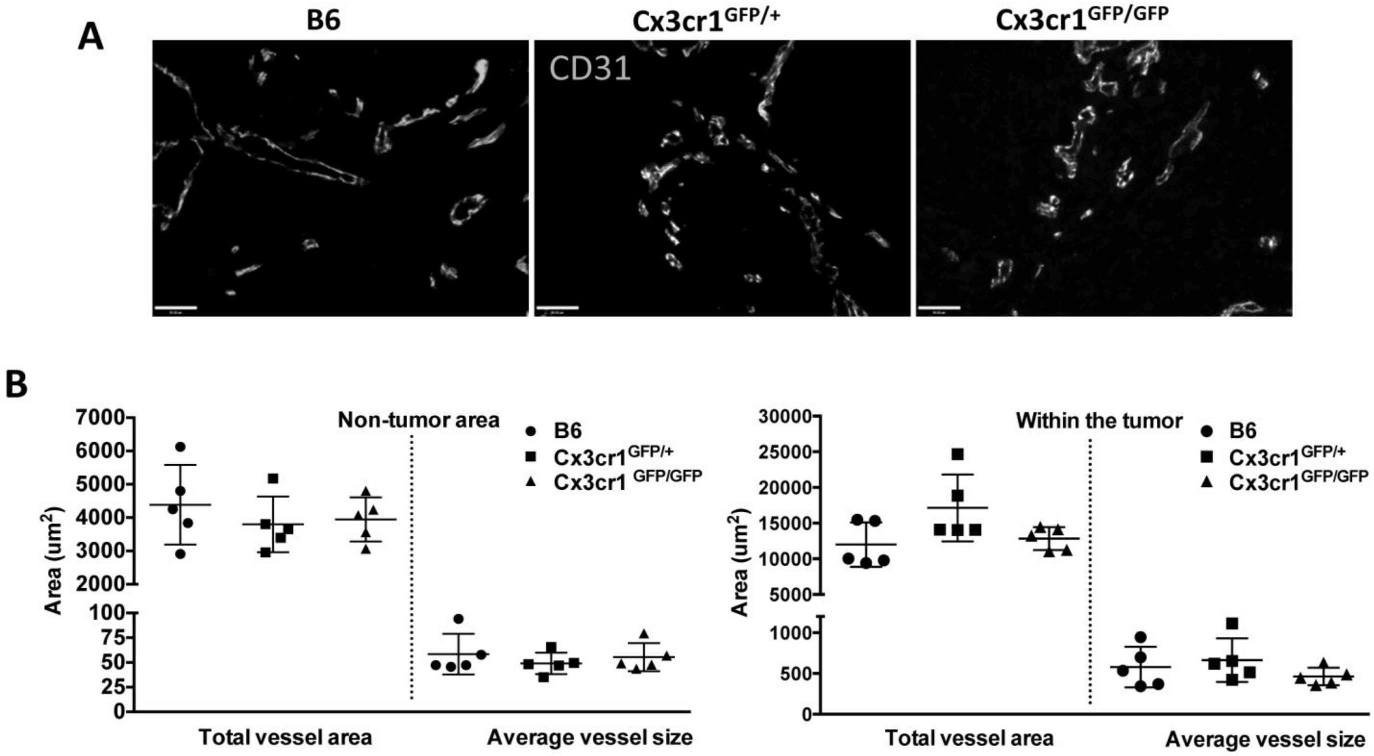

Figure S2: Neither heterozygous nor homozygous deletion of *Cx3cr1* impacts either total vessel area or average vessel size in the tumor or surrounding brain tissue. A) Representative images of CD31 staining in tumors from the three genotypes. **B)** Neither one nor two copies of *Cx3cr1* loss-of-function alleles impacted total vessel area or average vessel size (based on CD31 staining) in the surrounding non-tumor brain tissue or within the tumor in any of the three genotypes. Scale bar represents 50µm.

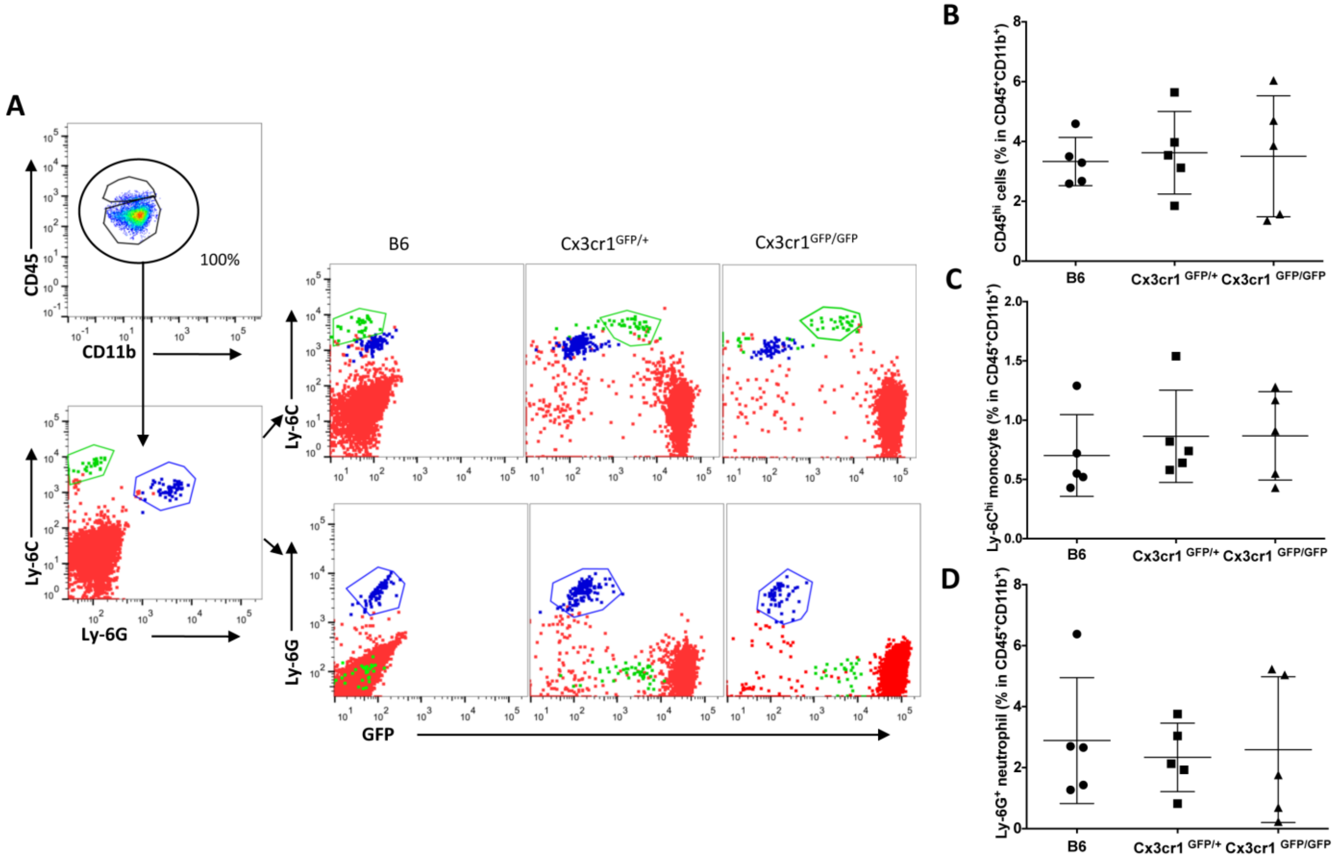

Figure S3: Loss of CX3CR1 has no impact on recruitment of Ly-6C^{hi} "inflammatory" monocyte into naïve brain. A) Representative dot plots that are gated on CD11b^{+}CD45^{+} cells, with red and green circles defining CD11b+CD45^{hi} (blood-derived macrophages) and CD11^{+}CD45 $^{lo/int}$ (resident brain microglia). Total population of CD11b^{+}CD45^{+} is considered as 100%, and they are further gated for Ly-6C and Ly-6G positivity to distinguish monocytes from neutrophils and further gated for GFP (CX3CR1), which shows that while inflammatory monocytes are positive for GFP, neutrophils are negative. **B)** Dot plots represent the percentage of CD45^{hi} population in the total CD11b^{+}CD45^{+} population of naïve brains from the three genotypes. There is no statistical significant difference in the % of CD45^{hi} cells in total CD11b^{+}CD45^{+} population (n=5 for *B6*, *Cx3cr1^{GFP+}* and *Cx3cr1$^{GFP/GFP}$*, respectively). **C)** Dot plots represent the percentage of Ly-6C^{hi} monocytes in the total CD11b^{+}CD45^{+} population in tumors from brains of three different genotypes (each dot corresponds to one animal). A one-way ANOVA with Tukey's multiple comparisons test was performed and demonstrated that there was no statistically significant increase in the percentage of Ly-6C^{hi} monocytes in brains from *Cx3cr1$^{GFP/GFP}$* animals compared to B6 or *Cx3cr1^{GFP+}* animals. **D)** Dot plots represent the percentage of Ly-6G^{+} neutrophils in brains from the three different genotypes showing that loss of one or both copies of *Cx3Cr1* had no impact on % of neutrophil infiltration into brain.

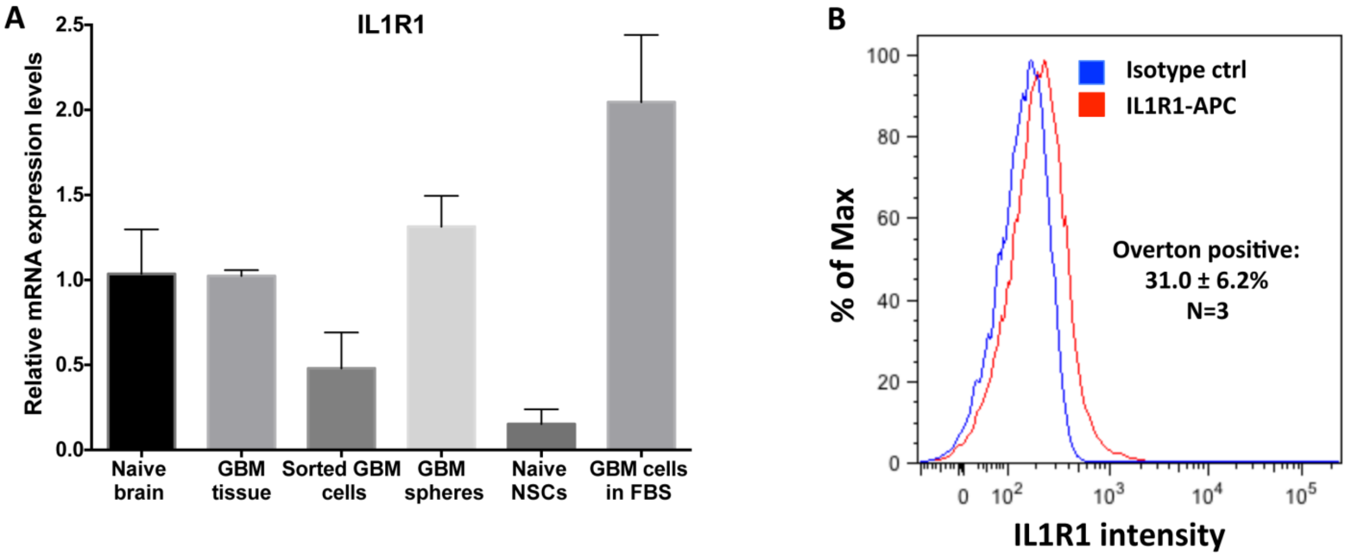

Figure S4: IL1R1 mRNA and protein are both expressed in GBM. A) Relative mRNA expression levels of IL1R1 show that GBM cells express the receptor (n=3 per each group). **B)** FACS analysis of freshly dissociated GBM cells reveled that these cells show a clear leftward shift in IL1R1 intensity compare to isotype control. Representative FACS pictures are from three independent GBM samples.

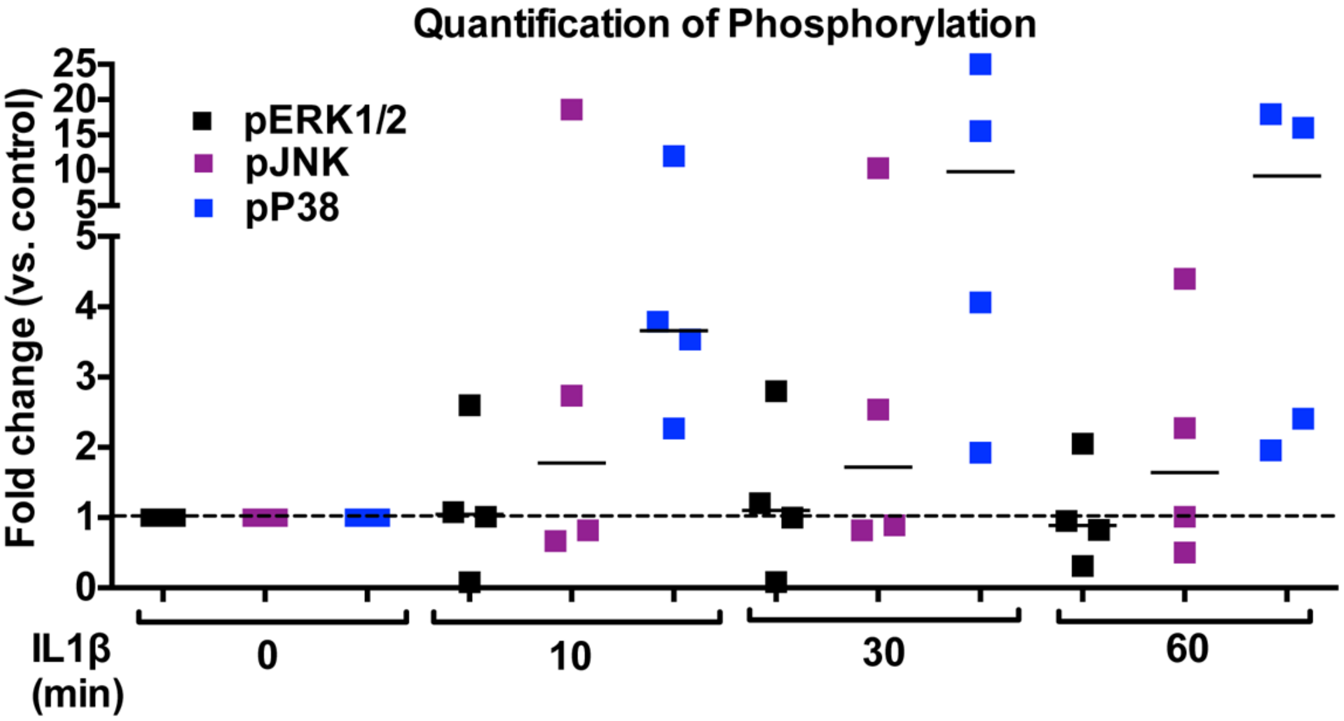

Figure S5: IL1β treatment induces activation of the p38 MAPK pathway in murine GSCs. Dot blots represent quantification of phosphorylation of ERK, JNK and 38 MAPK treated with 100pM IL1β at 0, 10, 30 and 60 minutes. Four primary GSC lines were tested and they all showed up-regulation of the p38 MAPK pathway.

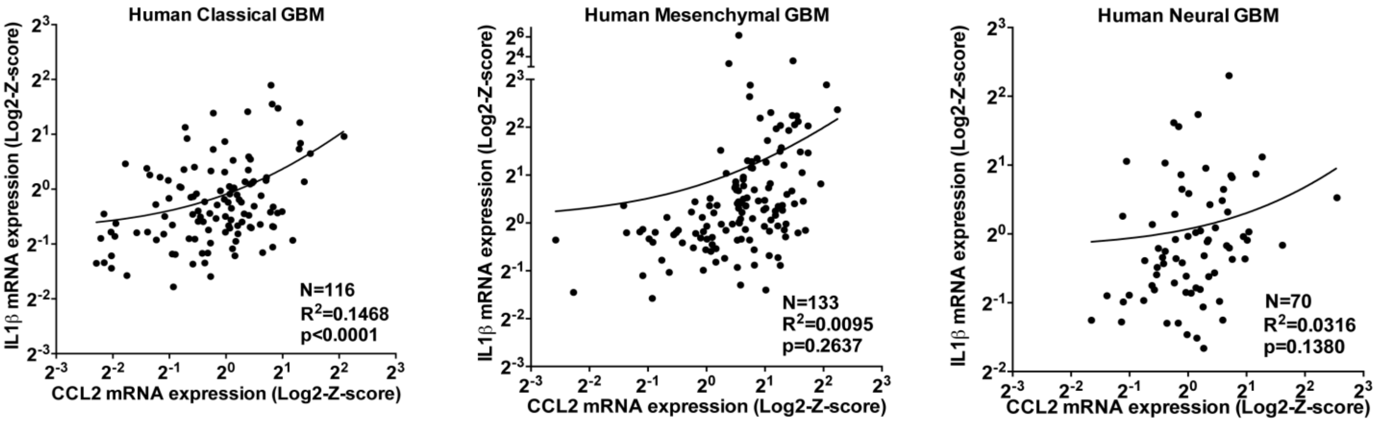

Figure S6: Graphs show correlations with linear regression for Classical, Mesemchymal, and Neural GBM samples for IL1β and CCL2 RNA expression from the TCGA database. A statistically significant positive correlation was found between IL1β and CCL2 RNA expression r=0.3831, p<0.0001. Linear regression R^2=0.1468, p<0.0001. No significant correlation was observed in Mesenchymal and Neural GBM.

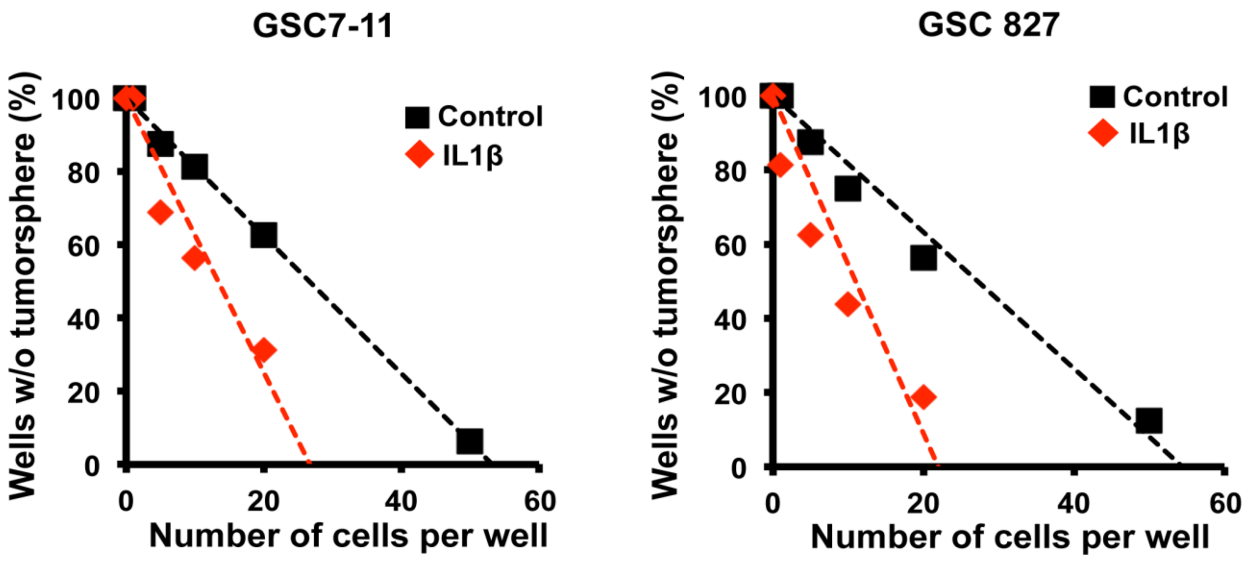

1/(Estimated stem cell frequency)		
Group	711	827
Control	29.6	26.6
IL1β	16	11.2

P value	
	Control vs IL1β
711	0.0153
827	0.000489

Figure S7: IL1β treatment enhances stem cell phenotype in human Proneural GSCs *in vitro.* Limiting dilution assay results for GSC7-11 and GSC 827 showing that there was a statistically significant increase in estimated stem cell frequency in response to IL1β treatment compared to control for both cell lines. Stem cell frequency and *p*-values were calculated using a web-based tool "ELDA" (extreme limiting dilution analysis), which is available on the Walter and Eliza Hall Institute of Medical Research website (http://bioinf.wehi.edu.au/software/elda/).

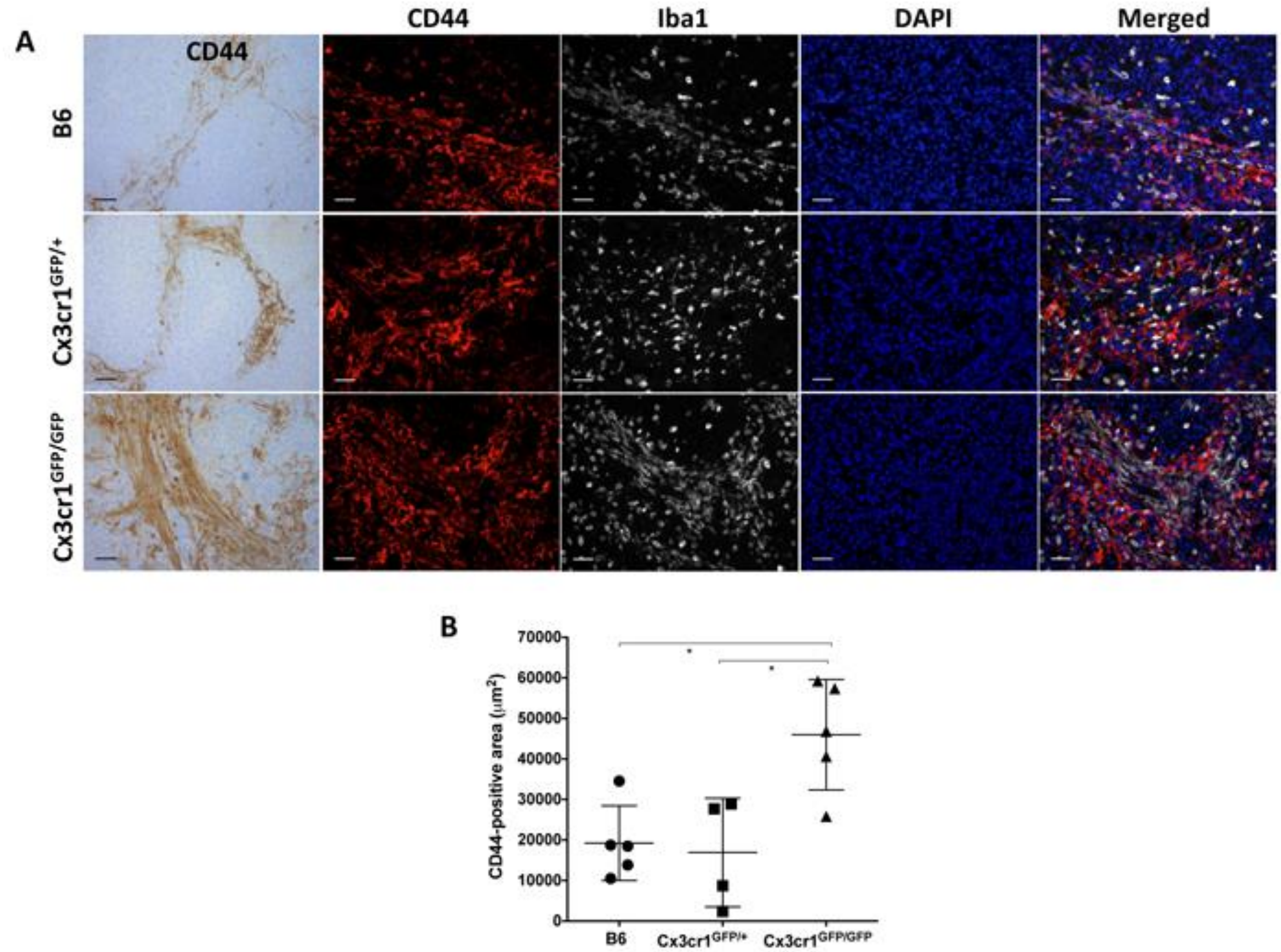

Figure S8: Loss of *Cx3Cr1* correlates with increased levels of CD44 positivity in PVA. A) Representative images of CD44 IHC in tumors generated in *B6*, *Cx3cr1*$^{GFP/+}$ and *Cx3cr1*$^{GFP/GFP}$ mice. Representative images of brain tumor sections from B6, *Cx3cr1*$^{GFP+}$ and *Cx3cr1*$^{GFP/GFP}$ mice were stained with anti-CD44 (red) and anti-Iba1 (grey) and were counterstained with nuclear DAPI (blue). **B)** Dot plots represent quantification of CD44-positive areas in tumors from the three genotypes (n=5 animals per each genotype) showed that loss of *Cx3cr1* results in a significant increase in CD44 in the PVA area (one-way ANOVA with Tukey's multiple comparisons test, *$p<0.05$). Scale bar represents 50 µm.

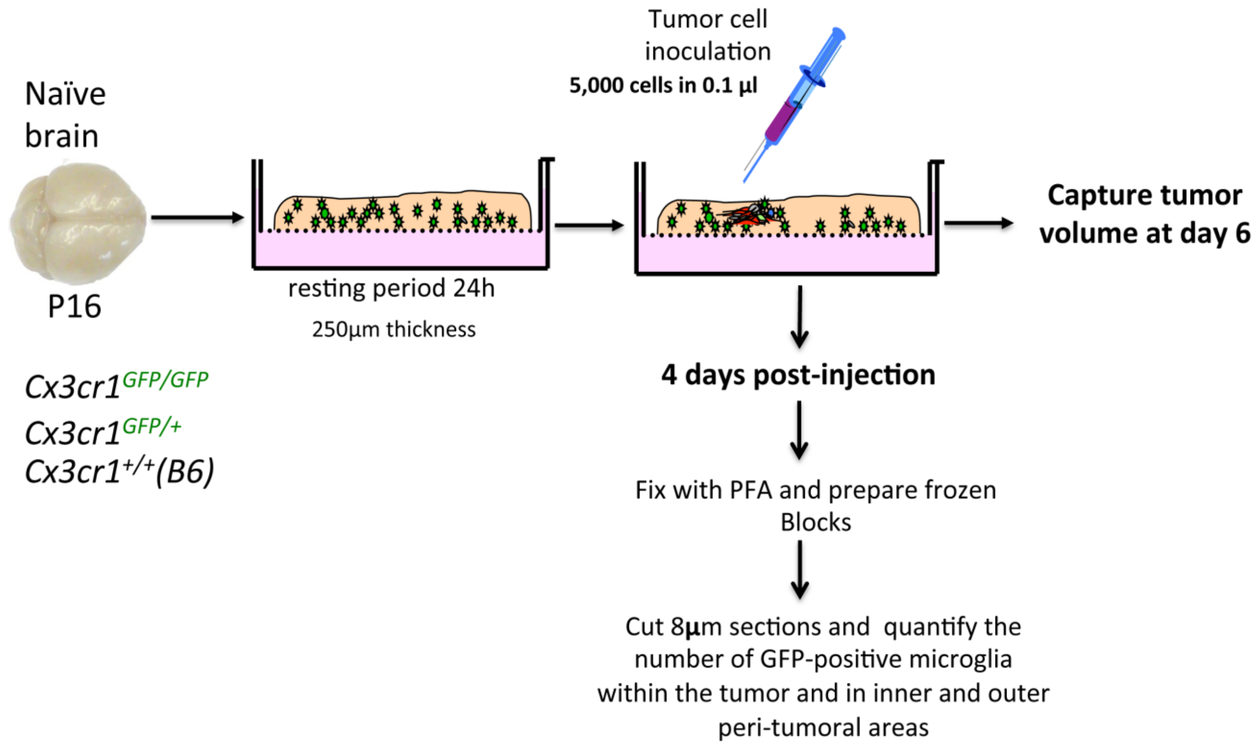

Figure S9: Illustration of the generation of gliomas in organotypic slice cultures *ex vivo.*

STable 1

A) List of Antibodies for Flow Cytometry

Description	Clone	Isotype	Fluorochrome	Company	Cat#
CD45	30-F11	Rat IgG2b,k	APC	Biolegend	103112
CD45	30-F11	Rat IgG2b,k	FITC	BD Pharmigen	553079
CD11b	M1/70	Rat IgG2b,k	PerCP-Cy5.5	BD Pharmigen	550993
F4/80	BM8	Rat IgG2a, k	PE	eBioscience	12-4801-80
F4/80	BM8	Rat IgG2a,k	APC	eBioscience	17-4801-80
Ly6C	AL21	Rat IgM,k	PE-Cy7	BD Pharmigen	560693
Ly6G	1A8	Rat IgG2a,k	V450	BD Horizon	560603
CD121a	JAMA-147	Hamster IgG	APC	Biolegend	113509
Hamster IgG ctrl	HTK888		APC	Biolegend	400911
Fc Block	2.4G2	Rat IgG2a,k	n/a	BD Pharmigen	553142
LIVE/DEAD staining kit	n/a	n/a	Aqua	Life technologies	L34957

B) Mouse Real-time qPCR primers

Gene name	Forward primer (5'-3')	Reverse Primer (5'-3')
**β**-actin	ACCCACACTGTGCCCATCTACG	GCCACGCTCGGTCAGGATCTTC
TNF-**α**	AAGCCTGTAGCCCACGTCGTA	GGCACCACTAGTTGGTTGTCTTTG
iNOS	CCCTTCAATGGTTGGTACATGG	ACATTGATCTCCGTGACAGCC
IL1**β**	CAGGCTCCGAGATGAACAAC	GGTGGAGAGCTTTCAGCTCATAT
CCL2	CCCACTCACCTGCTGCTACT	TCTGGACCCATTCCTTCTTG
Arg1	TGGGTGGATGCTCACACTGA	CAGGTTGCCCATGCAGATT
IL6	GCCTTCTTGGGACTGATGCT	AGTCTCCTCTCCGGACTTGTG
CD44	TCGATTTGAATGTAACCTGCCG	CAGTCCGGGAGATACTGTAGC
Mushashi	TAAAGTGCTGGCGCAATCG	TCTTCGTCCGAGTGACCATCT
IL1R1	GTTTCTGCTTTCACCACTCCA	GAGTCCAATTTACTCCAGGTCAG
Oct4	CGGAAGAGAAAGCGAACTAGC	ATTGGCGATGTGAGTGATCTG
Sox2	TCCAAAAACTAATCACAACAATCG	GAAGTGCAATTGGGATGAAAA
Nanog	CCTCAGCCTCCAGCAGATGC	CCGCTTGCACTTCACCCTTTG

Lee, J., S. Kotliarova, Y. Kotliarov, A. Li, Q. Su, N.M. Donin, S. Pastorino, B.W. Purow, N. Christopher, W. Zhang, J.K. Park, and H.A. Fine. 2006. Tumor stem cells derived from glioblastomas cultured in bFGF and EGF more closely mirror the phenotype and genotype of primary tumors than do serum-cultured cell lines. *Cancer cell* 9:391-403.
